# Transcriptional analysis of landmark molecular pathways in lung adenocarcinoma results in a clinically relevant classification with potential therapeutic implications

**DOI:** 10.1002/1878-0261.13550

**Published:** 2023-12-21

**Authors:** Sara Hijazo‐Pechero, Ania Alay, David Cordero, Raúl Marín, Noelia Vilariño, Ramón Palmero, Jesús Brenes, Aina Montalban‐Casafont, Ernest Nadal, Xavier Solé

**Affiliations:** ^1^ Unit of Bioinformatics for Precision Oncology, Catalan Institute of Oncology (ICO) L'Hospitalet de Llobregat Barcelona Spain; ^2^ Preclinical and Experimental Research in Thoracic Tumors (PrETT), Molecular Mechanisms and Experimental Therapy in Oncology Program (Oncobell), Bellvitge Biomedical Research Institute (IDIBELL) L'Hospitalet de Llobregat Barcelona Spain; ^3^ Translational Genomics and Targeted Therapies in Solid Tumors August Pi i Sunyer Biomedical Research Institute (IDIBAPS) Barcelona Spain; ^4^ Thoracic Oncology Unit, Department of Medical Oncology, Catalan Institute of Oncology (ICO) L'Hospitalet de Llobregat Barcelona Spain; ^5^ Neuro‐Oncology Unit, Catalan Institute of Oncology (ICO) L'Hospitalet de Llobregat Barcelona Spain; ^6^ Molecular Biology CORE, Center for Biomedical Diagnostics (CDB) Hospital Clínic de Barcelona Spain

**Keywords:** immunotherapy, lung adenocarcinoma, molecular pathways, precision oncology, transcriptional subtypes

## Abstract

Lung adenocarcinoma (LUAD) is a molecularly heterogeneous disease. In addition to genomic alterations, cancer transcriptional profiling can be helpful to tailor cancer treatment and to estimate each patient's outcome. Transcriptional activity levels of 50 molecular pathways were inferred in 4573 LUAD patients using Gene Set Variation Analysis (GSVA) method. Seven LUAD subtypes were defined and independently validated based on the combined behavior of the studied pathways: AD (adenocarcinoma subtype) 1–7. AD1, AD4, and AD5 subtypes were associated with better overall survival. AD1 and AD4 subtypes were enriched in epidermal growth factor receptor (*EGFR*) mutations, whereas AD2 and AD6 showed higher tumor protein p53 (*TP53*) alteration frequencies. AD2 and AD6 subtypes correlated with higher genome instability, proliferation‐related pathway expression, and specific sensitivity to chemotherapy, based on data from LUAD cell lines. LUAD subtypes were able to predict immunotherapy response in addition to *CD274* (PD‐L1) gene expression and tumor mutational burden (TMB). AD2 and AD4 subtypes were associated with potential resistance and response to immunotherapy, respectively. Thus, analysis of transcriptomic data could improve patient stratification beyond genomics and single biomarkers (i.e., PD‐L1 and TMB) and may lay the foundation for more personalized treatment avenues, especially in driver‐negative LUAD.

## Graphical abstract

We classified lung adenocarcinoma (LUAD) tumors into seven subtypes, based on the transcriptional activity of 50 pathways. Subtypes were associated with distinct molecular and clinical features. Despite significant challenges, we believe that integration of transcriptomic and genomic data may pave the way for guiding novel therapeutic approaches in patients with LUAD.

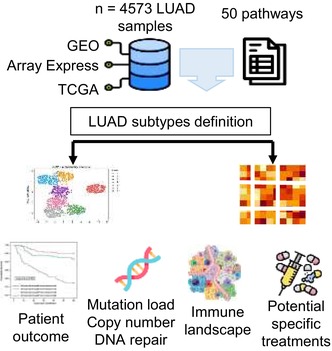

**Abbreviations**
AAC: area above the curve
CNA: copy number alteration
DDR: DNA damage repair
FDR: false discovery rate
GEO: Gene Expression Omnibus
GSVA: gene set variation analysis
ICA: immune checkpoint activators
ICI: immune checkpoint inhibitors
LUAD: lung adenocarcinoma
LUAD‐CCL: lung adenocarcinoma cancer cell lines
NSCLC: non‐small cell lung cancer
OS: overall survival
SNV: somatic single nucleotide variants
TMB: tumor mutational burden
TPM: transcripts per million
UMAP: Uniform Manifold Approximation and Projection
WES: whole‐exome sequencing
WHO: World Health Organization

## 1 Introduction

Lung cancer is a major global health problem. According to the World Health Organization (WHO), lung cancer was the leading cause of cancer‐related deaths and the second most frequently diagnosed cancer in 2020 [1]. Regarding histological subtypes, lung adenocarcinoma (LUAD) is the most prevalent histological entity, accounting for almost 55% of the diagnoses [2]. In terms of clinical management, chemotherapy alone or in combination with immunotherapy is considered the standard of care for patients with advanced LUAD not harboring actionable oncogenic alterations [3]. Additionally, recent advances in high‐throughput genomic technologies for molecular profiling have accelerated the evolution of personalized medicine [4, 5]. For instance, the current management of LUAD requires molecular testing to detect actionable genomic alterations predicting clinical benefit to targeted therapies [3]. However, patients with advanced LUAD have heterogeneous responses and poor survival outcomes (5‐year survival rate = 21%) [6]. These differences between patient response rates have been attributed to tumor burden, comorbidities, functional status, or tumor heterogeneity, such as different immune landscapes, activation of signaling pathways, and presence of different cell types [7]. Thus, improving LUAD patients' stratification beyond genomic testing could move forward precision medicine, but is a major challenge.

Given the limitations of genomics to capture the complexity of LUAD and to predict response to specific treatments, innovative approaches are needed to improve clinical outcome. In this regard, gene expression profiling has already been used to further stratify LUAD into different molecular subtypes [8]. However, the clinical relevance of those classifications was questioned due to technical intrinsic limitations, inconsistencies between studies, and the lack of association with potential therapeutic strategies.

The aim of our study was to develop a novel LUAD classification based on transcriptomics able to improve patients' stratification beyond the current histological and genomic‐based classifications. For this purpose, we integrated transcriptional profiles from more than 4500 LUAD. To the best of our knowledge, this is the largest study defining transcriptional LUAD subtypes [8]. In addition, unlike previous attempts relying on measuring individual gene expression, we assessed the activity of a set of well‐defined molecular pathways, which makes it less prone to variability [9, 10]. Based on this, a computational framework was developed to stratify LUAD into different subtypes based on the expression of specific signaling pathways. These subtypes were further characterized at different levels (i.e., clinical covariates, genomic features, and immune landscape). Finally, the analysis of publicly available large‐scale cancer cell line drug screening projects revealed potential therapeutic vulnerabilities for each group of LUAD tumors [11, 12, 13, 14]. Overall, this classification may delineate novel therapeutic strategies beyond current genomic‐based targeted therapies, which could be especially relevant in the case of driver‐negative LUAD patients.

## 2 Materials and methods

### 2.1 Datasets and gene expression data processing

LUAD transcriptional profiles were obtained from Gene Expression Omnibus (GEO), Lung Cancer Explorer, and ArrayExpress public data archives [15, 16, 17]. Subsequent filters were applied to keep human LUAD tumor samples, exclude datasets with less than 10 samples, and remove those studies using platforms that do not cover a significant part of the transcriptome (i.e., targeted panels covering a smaller subset of genes). Overall, 56 datasets were included in this analysis, constituting more than 4500 LUAD samples (Table S1, Fig. S1).

Raw transcriptomics data were downloaded when available and later processed using the recommended method for each microarray platform (i.e., Affymetrix (Santa Clara, CA, USA), Agilent (Santa Clara, CA, USA), and Illumina (San Diego, CA, USA).

#### 2.1.1 Affymetrix platforms data processing

Raw expression data from two‐color Affymetrix platforms (Table S1) were processed using robust multiarray average algorithm (RMA) implemented in the affy package version 1.56 available through the bioconductor software project (https://bioconductor.org).

Probeset‐to‐gene mapping was done using BioMart web services via biomart r package version 2.34 [18], selecting the most expressed probe as representative of gene expression when multiple mapping probes occurred to avoid duplicated genes.

#### 2.1.2 Two‐color Agilent and CHUGAI platforms data processing

Raw expression data from two‐color Agilent and CHUGAI platforms (Table S1) were processed using minimum background correction method as implemented in the *backgroundCorrection* function of the limma package available in r (https://www.r‐project.org/). Background correction accounts for possible biases related to non‐specific binding or spatial heterogeneity across the array. The next step in the normalization process is correcting for dye biases due to the presence of two colors in the array. This correction was performed using the loess method from the *normalizeWithinArrays* function also included in the limma package. This method returns a matrix of corrected *M* and *A* values using the following expressions:

$$M=\log_{2}\left(R/G\right)=\log_{2}\left(R\right)-\log_{2}\left(G\right),$$

$$A=\frac{1}{2}{\text{log}}_{2}\left(\mathrm{RG}\right)=\frac{1}{2}\left(\log_{2}\left(R\right)+\log_{2}\left(G\right)\right).$$

The idea is to scale the log‐ratios to have the same median absolute deviation (MAD) across samples. After normalizing each sample for dye biases, a normalization step between samples is needed to make them comparable with each other. This is achieved using the quantile method of the *normalizeBetweenArrays* function within the R limma package. Finally, the normalized intensity values for the sample channel (i.e., red or green depending on the array design) are retrieved by solving the above‐mentioned expressions, using the already calculated and normalized *M* and *A* values.

The probe‐to‐gene annotation was performed using the r package biomart version 2.34. When multiple probes mapped to the same gene, the most expressed one was selected to obtain a single representative probe for each gene. Then, hgnchelper package was used for the identification and correction of obsolete or invalid gene symbols to harmonize all datasets.

#### 2.1.3 Illumina Beadchip Platforms data processing

Raw expression data from Illumina BeadChip Platforms (Table S1) were processed using the RMA background correction method as implemented in the *backgroundCorrection* function of the limma package. Secondly, since this is a single‐channel platform there is no need to perform a within‐sample normalization, although between‐sample normalization is still required. In this case, this is achieved using the quantile normalization method of the *normalizeBetweenArrays* function within the limma package. The quantile approach makes the distribution of microarray signals the same between all arrays, making samples comparable between them. Then, hgnchelper package was used for the identification and correction of obsolete or invalid gene symbols to harmonize all datasets.

The probeset‐to‐gene annotation was performed using the R package biomart version 2.34. When multiple probes mapped to the same gene, the most expressed one was selected to obtain a single representative probe for each gene. Then, hgnchelper package was used for the identification and correction of obsolete or invalid gene symbols to harmonize all datasets.

For the case of TCGA‐LUAD RNA‐seq dataset, transcripts per million processed data were downloaded from TCGA2BED FTP repository [19].

### 2.2 LUAD consensus pathway transcriptional subtype definition framework

LUAD consensus transcriptional subtype classification framework is depicted in Fig. S2. Briefly, Gene Set Variation Analysis (GSVA) algorithm was used to evaluate the activity level of the 50 pathways included in the MSigDB hallmarks collection in each dataset, using a *k*‐fold approach (*k* = 5) across 100 iterations [9, 10]. Uniform Manifold Approximation and Projection (UMAP) dimension reduction method and walktrap clustering (Euclidean distance) were subsequently conducted on the previously obtained GSVA scores matrices to identify potential LUAD subpopulations [20]. Summary metrics for each potential LUAD subpopulation were calculated and used to establish final LUAD consensus subtypes using UMAP and walktrap method. Finally, tumor samples were assigned to the subtype to which they had been assigned the majority of times across the classification framework.

### 2.3 LUAD molecular subtype characterization

#### 2.3.1 Clinicopathological covariates and overall survival

Association with clinicopathological variables (e.g., age, sex, stage, smoking status, and presence/absence genomic alterations) was assessed using comparegroups package for R (V.4.2.0) [21]. Data regarding the presence/absence of LUAD oncogenic alterations (e.g., *EGFR*, *KRAS*, *ALK*, *TP53*, and *STK11*) were collected from the clinical data of the datasets included in this study when available.

The Cox proportional hazards models adjusted for age, sex, stage, smoking status, and study were used to test for the impact of our classification on overall survival (OS) rate.

#### 2.3.2 Genomic characterization

TCGA‐LUAD dataset [18] had available somatic alterations data for evaluating tumor mutational burden (TMB) and COSMIC v3 mutational signatures [22]. For TMB, the total number of alterations per sample was assessed excluding synonymous variants. These values were then divided by the number of megabases (Mb) covered by the TCGA‐LUAD whole‐exome sequencing (WES) panel to obtain the number of mutations per Mb or TMB. Using somatic single nucleotide variants (SNV), mutational signatures were inferred using the R package sigprofilerextractorr [23].

Copy number alteration (CNA) levels were also evaluated in the TCGA‐LUAD dataset [18]. Finally, genome instability was assessed using previously calculated DNA damage repair (DDR) deficiency scores in the TCGA‐LUAD dataset [24].

#### 2.3.3 Impact of the LUAD molecular classification on the immune landscape and immunotherapy response

The immune infiltrate composition of each LUAD sample was inferred using GSVA algorithm [10]. Gene signatures of the 21 evaluated immune fractions were obtained from a previous study [25]. Due to GSVA methodological constraints, single‐gene signatures were replaced by their multi‐gene counterparts published in a different study [26]. In addition, we also used specific cell categories when available, instead of the more generic supercategory (i.e., *M1 macrophages* and *M2 macrophages* instead of the broader *macrophages* category). For each cell type, we calculated the percentage of enriched tumors. Median GSVA scores for each cell fraction were used as the cut‐off to define whether a sample is enriched in a specific cell type.

The status of a set of immune checkpoint inhibitors (ICI), activators (ICA), and T‐cell effector and exhaustion markers was also evaluated [27, 28]. For each gene expression dataset, the median gene expression value of each marker was used as the cut‐off point for deciding whether a sample is enriched for a specific immune biomarker.

The predicted response to immunotherapy treatment was derived from the Tumor Immune Dysfunction and Exclusion (TIDE) scores already calculated for TCGA‐LUAD dataset [29]. TIDE‐positive scores indicate that a sample is less likely to respond to immunotherapy, because of the presence of immunosuppressive signals, whereas negative scores indicate potential response to immune checkpoint treatment (i.e., anti‐CTLA4 and anti‐PD‐1). Binomial generalized linear models adjusted for PD‐L1 gene expression and TMB values were used to test the impact of our classification on potential immunotherapy response.

### 2.4 Consensus transcriptional subtype independent validation

Subtyping of new samples in the CPTAC‐3 validation cohort was inferred using the *predict* function of the umap r package version 0.2.7.0 and a *k*‐nearest‐neighbors approximation [20, 30]. In summary, for each sample we obtained GSVA scores of the same 50 molecular pathways used to establish the original classification of LUAD tumors. This step was performed following the same steps previously described for the LUAD consensus pathway transcriptional subtype definition (fivefold, 100 iterations). Then, for each iteration, these GSVA scores were passed as an input to the *predict* function that produces 2D coordinates to map new samples onto the consensus map of LUAD tumors. New samples' subtype was predicted based on the most frequent label of the closest neighbors in the original classification. Therefore, after 100 iterations, each validation sample had 100 putative group assignations. Finally, samples were allocated to the AD subtype to which they had been assigned the majority of times throughout the classification process.

### 2.5 Identification of potential therapeutic vulnerabilities

Drug sensitivity data from three large pharmacogenomics studies were integrated to identify potential therapeutic vulnerabilities for each subtype using pharmacogx bioconductor/r package [31]. First, LUAD cancer cell lines (LUAD‐CCL) were classified based on the primary tumor's classification using the *predict* function within umap r package as previously described for the cancer cell lines classification. Area above the curve (AAC) sensitivity measures for each drug and cell line were used to identify potential therapeutic vulnerabilities for the different subtypes. Importantly, *PharmacoGx* AAC values were normalized by the concentration range of the experiment in each study and take values between [0, 1]. Thus, the greater the AAC the more effective is a drug against a specific cell line. Subtypes were considered as potentially sensitive to the treatment if the average AAC value for the cell lines classified within a certain group was greater than the mean AAC plus 2 standard deviations for the drugs assessed in at least 2 out of the 3 pharmacogenomics studies. Also, average AACs were only calculated if the treatment had been tested in at least 2 different cell lines within a subtype and study.

## 3 Results

### 3.1 Consensus classification based on expression of 50 landmark molecular pathways yielded seven transcriptional LUAD subtypes

**Fig. 1:**
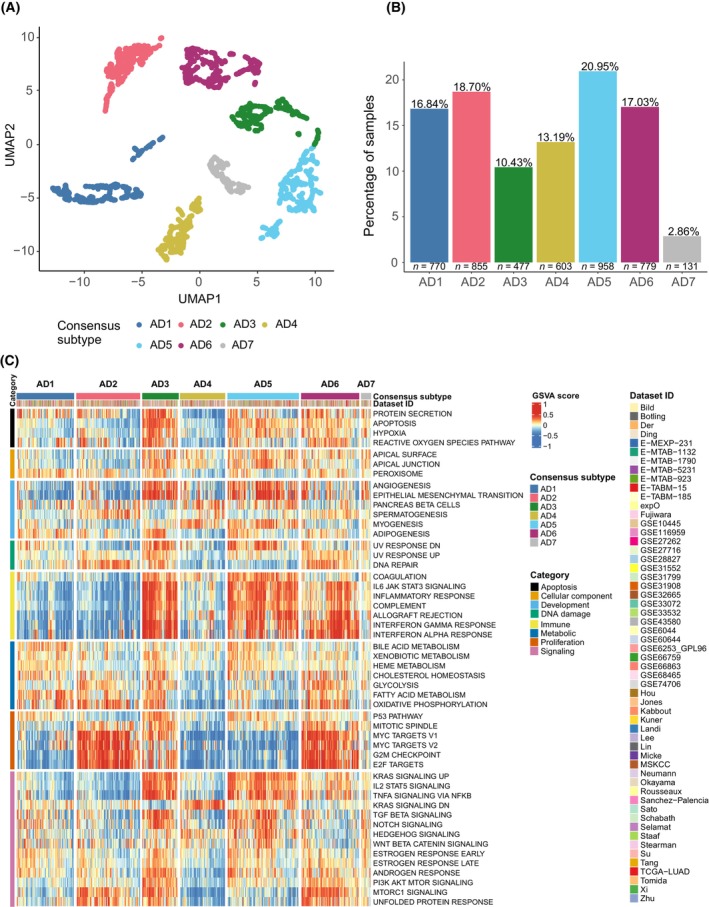
LUAD subtype pathway transcriptional landscape. (A) LUAD consensus map of pathway transcriptional profiling‐based subpopulations. Each dot represents the summary centroid of the different subpopulations identified during the classification process. Using UMAP and walktrap clustering method with Euclidean distance on these centroids, seven different consensus groups, represented by different colors, were identified based on the joint behavior of the 50 studied molecular pathways. (B) Barplots representing the distribution of LUAD tumors across the seven transcriptional subtypes. (C) Heatmap representing relative activity levels (GSVA scores) of the 50 studied pathways (rows) in each of the 4573 LUAD tumor samples (columns) that were assigned to a consensus subtype. Red colors indicate higher relative activity of a pathway in a certain sample, whereas blue colors indicate lower relative activity of a pathway in a certain sample.

**Fig. 2:**
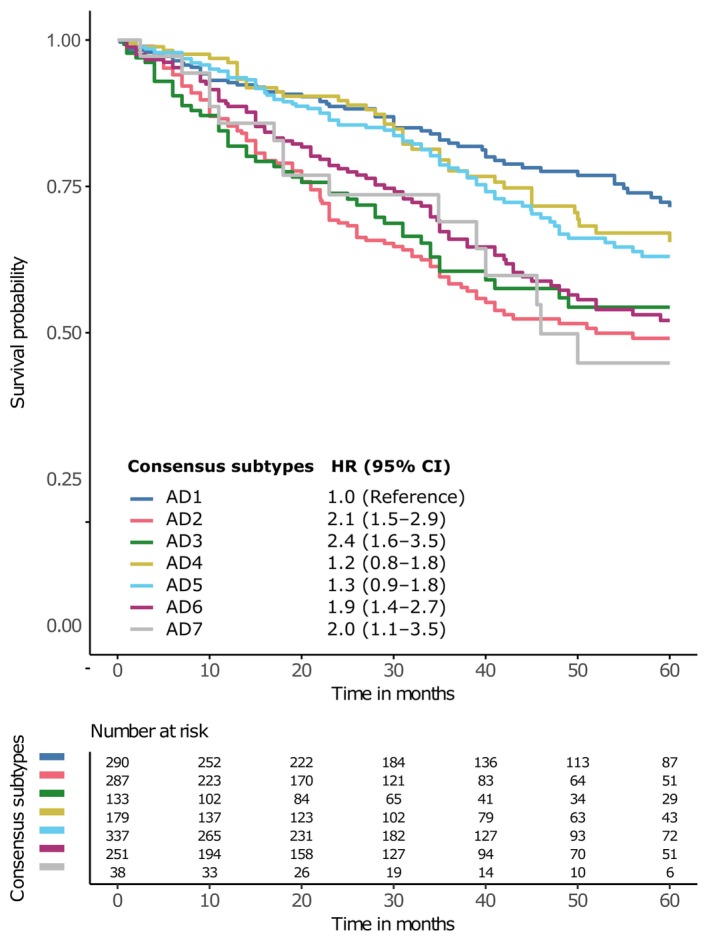
Overall survival by LUAD subtype. Kaplan–Meier curves of each of the identified pathway transcriptional profiling‐based LUAD groups. Hazard ratios (HR) and 95% confidence intervals (95% CI) come from a Cox proportional hazards model adjusted for age, sex, stage, smoking history, and dataset. For this analysis, we used the subset of datasets with available survival data and complete covariates information for the Cox proportional hazards model (*n* = 10 datasets, *n* = 1515 samples).

GSVA was conducted on more than 4500 LUAD in order to establish a consensus transcriptional classification based on the activity levels of 50 signaling pathways (see Section 2; Table S1) [9, 10]. Using this approach, we identified seven LUAD transcriptional‐based subtypes, labeled as AD1‐7 (Fig. 1A). These subtypes were not evenly distributed throughout the whole set of tumors analyzed in this study (Fig. 2B). The most represented subtype was AD5 accounting for 20.95% of the tumors, whereas AD7 represented only 2.86% of the tumors.

Based on the relative activity of the signaling molecular pathways, each group displayed a specific transcriptional fingerprint (Fig. 1C, Fig. S3). A summary of the relatively upregulated and downregulated pathways within each LUAD subtype is depicted in Table 1.

**Table 1:** Molecular pathway landscape across LUAD subtypes.

Consensus subtype	Upregulated pathways	Downregulated pathways
AD1	Metabolic pathways	Angiogenesis Epithelial–mesenchymal transition Immune‐related pathways Cell cycle‐related pathways PI3K‐AKT–MTOR signaling
AD2	DNA repair Oxidative phosphorylation Cell cycle‐related pathways	Angiogenesis Epithelial–mesenchymal transition Immune‐related pathways Apoptosis TGF‐B signaling Hedgehog/Notch signaling IL2‐STAT5 signaling
AD3	Angiogenesis Epithelial–mesenchymal transition Immune‐related pathways Metabolic pathways Apoptosis Hypoxia Protein secretion TP53 pathway KRAS signaling IL2‐STAT5 signaling TNFA via NFKB signaling PI3K‐AKT–MTOR signaling TGF‐B signaling Notch signaling MTORC1 signaling	
AD4		DNA repair Metabolic pathways Apoptosis Hypoxia Protein secretion Cell cycle‐related pathways KRAS signaling PI3K‐AKT–MTOR signaling TGF‐B signaling MTORC1 signaling Unfolded protein response
AD5	Immune system‐related pathways Apoptosis TP53 pathway IL2‐STAT5 signaling TNFA via NFKB signaling Hedgehog signaling	DNA repair Metabolic pathways Cell cycle‐related pathways Unfolded protein response
AD6	DNA repair Interferon‐gamma Interferon alpha Metabolic pathways Cell cycle‐related pathways PI3K‐AKT–MTOR signaling MTORC1 signaling Unfolded protein response	Hedgehog signaling WNT B‐catenin signaling
AD7	Estrogen response Notch signaling	

### 3.2 LUAD transcriptional subtypes are correlated with clinicopathological covariates, distinct genomic profile, and overall survival

We evaluated the correlation of these subgroups with clinicopathological characteristics and whether they are represented across all the datasets included in the study (Table 2, Table S2). We observed a significant association with all evaluated covariates. Subtypes were represented in the different studies, although some subtypes may be more represented and underrepresented in certain datasets, most likely due to intrinsic biases of retrospective studies.

**Table 2:** Correlation of clinicopathological variables with LUAD subtypes. The number of samples with available information in each case is depicted in the *N* column. MUT, mutated; WT, wildtype.

	*N*	AD1 (%)	AD2 (%)	AD3 (%)	AD4 (%)	AD5 (%)	AD6 (%)	AD7 (%)	*P*
		*N* = 766	*N* = 851	*N* = 473	*N* = 598	*N* = 952	*N* = 774	*N* = 129	
Sex, *N* (%)	3906								< 0.001
M		324 (50.47)	427 (56.86)	217 (51.91)	223 (42.72)	356 (43.41)	331 (50.46)	48 (49.48)	
F		318 (49.53)	324 (43.14)	201 (48.09)	299 (57.28)	464 (56.59)	325 (49.54)	49 (50.52)	
Age, *N* (%)	3609								< 0.001
≤ 50		55 (9.18)	77 (11.16)	33 (8.62)	34 (7.02)	61 (8.07)	61 (10.13)	14 (14.74)	
> 50–65		245 (40.90)	325 (47.10)	148 (38.64)	198 (40.91)	282 (37.30)	271 (45.02)	41 (43.16)	
> 65		299 (49.92)	288 (41.74)	202 (52.74)	252 (52.07)	413 (54.63)	270 (44.85)	40 (42.11)	
Stage, *N* (%)	3128								0.005
Early‐stage (I–II)		472 (86.61)	493 (80.42)	260 (81.00)	319 (85.07)	578 (85.50)	408 (79.53)	67 (78.82)	
Late‐stage (III–IV)		73 (13.39)	120 (19.58)	61 (19.00)	56 (14.93)	98 (14.50)	105 (20.47)	18 (21.18)	
Smoking history, *N* (%)	2788								< 0.001
Never smoker		152 (30.83)	64 (12.19)	56 (20.29)	99 (26.83)	164 (27.89)	81 (17.16)	16 (24.62)	
Smoker		341 (69.17)	461 (87.81)	220 (79.71)	270 (73.17)	424 (72.11)	391 (82.84)	49 (75.38)	
*EGFR* mutation, *N* (%)	1537								< 0.001
WT		185 (62.93)	231 (79.66)	105 (76.09)	111 (63.43)	248 (71.06)	195 (77.38)	24 (61.54)	
MUT		109 (37.07)	59 (20.34)	33 (23.91)	64 (36.57)	101 (28.94)	57 (22.62)	15 (38.46)	
*KRAS* mutation, *N* (%)	1360								0.239
WT		184 (72.73)	173 (70.90)	79 (63.71)	124 (78.48)	225 (72.12)	169 (73.48)	28 (71.79)	
MUT		69 (27.27)	71 (29.10)	45 (36.29)	34 (21.52)	87 (27.88)	61 (26.52)	11 (28.21)	
*ALK* translocation, *N* (%)	456								0.064
WT		99 (94.29)	67 (83.75)	24 (85.71)	52 (85.25)	83 (91.21)	76 (96.20)	10 (83.33)	
MUT		6 (5.71)	13 (16.25)	4 (14.29)	9 (14.75)	8 (8.79)	3 (3.80)	2 (16.67)	
*TP53* mutation, *N* (%)	849								< 0.001
WT		128 (85.91)	83 (50.30)	65 (78.31)	69 (75.00)	171 (85.93)	80 (56.34)	14 (73.68)	
MUT		21 (14.09)	82 (49.70)	18 (21.69)	23 (25.00)	28 (14.07)	62 (43.66)	5 (26.32)	
*STK11* mutation, *N* (%)	598								< 0.001
WT		80 (75.47)	87 (73.11)	53 (92.98)	63 (92.65)	128 (91.43)	87 (91.58)	12 (92.31)	
MUT		26 (24.53)	32 (26.89)	4 (7.02)	5 (7.35)	12 (8.57)	8 (8.42)	1 (7.69)	

We also evaluated the association of the LUAD subtypes with the presence of clinically relevant driver oncogenic alterations (Table 2). *EGFR* mutations occurred more frequently in AD1, AD4, and AD7 groups, while *TP53* mutations were more common in AD2 and AD6 subtypes, and *STK11* alterations were enriched in AD1 and AD2 subtypes. *KRAS* mutations and *ALK* rearrangements were not correlated with any of the subgroups.

We also assessed whether this classification was associated with overall survival (OS) (Fig. 2, Fig. S4). AD1, AD4, and AD5 patients were associated with longer OS whereas, overall, AD2, AD3, AD6, and AD7 showed worse survival outcomes. This analysis was adjusted for the following covariates: age, gender, tumor stage, smoking history, and dataset.

### 3.3 LUAD pathway transcriptional profiling‐based subtypes further subdivide previous mRNA‐based subtypes

We performed a comparison with the previous LUAD mRNA‐based consensus classification (bronchioid, squamoid, and magnoid) first described by Hayes et al. and later adopted by Wilkerson et al. and the TCGA for further exploration [18, 32, 33] (Fig. S5). Bronchioid mRNA subtype better aligned with AD1, AD4, and AD5 subtypes, all of them showing lower expression of proliferation‐related pathways (Fig. 1C). AD1, AD4, and AD5 had consistently better OS as described for bronchioid tumors, when compared to squamoid or magnoid subtypes. Also, bronchioid subtype was enriched for *EGFR* mutations, which was also observed in AD1 and AD4 subtypes. Squamoid mRNA subtypes were for the most part associated with AD3, AD5, and AD6 subtypes, all of them showing higher expression of immune‐related functions (Fig. 1C). This correlates with the higher immune cells infiltration previously found for squamoid tumors [34]. Moreover, AD6 was found to be enriched in *TP53* mutations; a trait also described for squamoid mRNA subtype. Finally, magnoid subtype mainly overlapped with AD2 proliferative subtype, which was enriched for *TP53/STK11* mutations (Fig. 1C, Table 2). Overall, these results demonstrate concordance among both LUAD classifications, but previous mRNA‐based subtypes were further subdivided by using our approach.

### 3.4 LUAD transcriptional subtypes were also correlated with tumor mutational burden and DNA damage

**Fig. 3:**
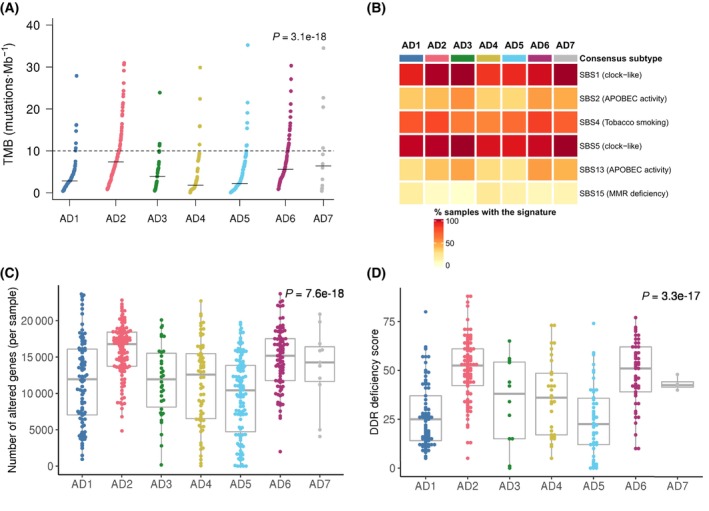
Genomic characterization in the TCGA‐LUAD set. (A) Tumor mutational burden (TMB) across LUAD consensus subtypes. Each dot represents the TMB value for a specific sample. The black segment represents the median TMB value for each LUAD subtype. The horizontal dotted line represents 10 mutations·Mb^−1^ TMB value, which is a common cut‐off for designating TMB high or low. Kruskal–Wallis test was used to assess potential differences regarding TMB between LUAD subtypes. *P* value was corrected using the false discovery rate (FDR) multiple‐comparison correction method. (B) Heatmap representing the percentage of positive samples for each specific COSMIC mutational signature (rows) in each LUAD subtype (columns). Samples were designated as positive if they harbored at least one mutation associated with a certain mutational signature. (C) Boxplots of the copy number alterations burden across LUAD subtypes. Each dot represents the number of altered genes per sample. Kruskal–Wallis test was used to assess potential differences regarding the number of copy number altered genes between LUAD subtypes. *P* value was corrected using the false discovery rate (FDR) multiple‐comparison correction method. (D) Boxplots of the DNA damage repair (DDR) deficiency score distribution across LUAD subgroups. Each dot represents the DDR score per sample. Kruskal–Wallis test was used to assess potential differences regarding DDR scores between LUAD subtypes. *P* value was corrected using the false discovery rate (FDR) multiple‐comparison correction method. For A–D, only the TCGA‐LUAD dataset was used as it is the only one with associated transcriptomics and genomics data. Number of samples of each subtype are: AD1: 90, AD2: 114, AD3: 34, AD4: 66, AD5: 113, AD6: 85, AD7: 12.

Using the TCGA‐LUAD dataset, LUAD subtypes were further characterized at the genomic level [18]. First, using whole‐exome sequencing data we evaluated potential differences in terms of TMB and mutational signatures included in the COSMIC v3 collection (Fig. 3A,B) [22]. TMB significantly differed among LUAD transcriptional subtypes (Fig. 3A). AD2 and AD6, which are also enriched for *TP53* mutations, had significantly higher TMB values when compared to the rest of subtypes, except for AD7 (Table S3). Concerning COSMIC mutational signatures, tobacco and clock‐like signatures were overrepresented across LUAD subtypes (Fig. 3B). Notably, our results showed a significant association between the subtypes and the prevalence of mutational signatures SBS1 (clock‐like), SBS4 (tobacco), and SBS13 (APOBEC activity) (Table S4).

Using TCGA‐LUAD data [18], copy number alterations (i.e., amplifications or deletions) were more common in AD2 and AD6 subtypes compared to the rest of the subtypes, except for AD7 (Fig. 3C, Table S5). We also assessed the level of genomic instability and DNA damage repair (DDR) capacity according to these subgroups (Fig. 3D). Again, AD2 and AD6 samples showed significantly higher DDR deficiency scores than the rest of subtypes (Table S6).

### 3.5 LUAD molecular subtypes had distinct immune cells infiltration patterns and were associated with different immunotherapy responses

**Fig. 4:**
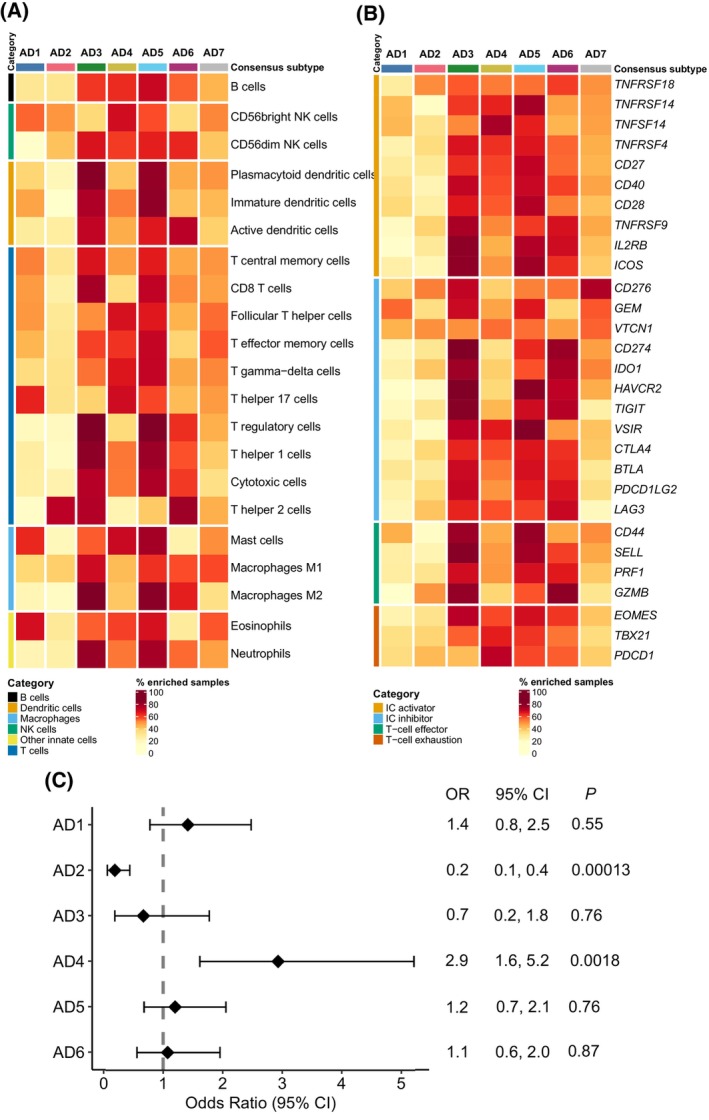
Immune characterization and association with immunotherapy response. (A) Heatmap representing the percentage of samples showing high relative infiltration of 21 evaluated immune cell types. Median immune cell abundance GSVA score values were used as a cut‐off to designate if a sample is enriched for a specific immune cell. Different immune cell categories are represented with different colors on the left side of the heatmap. (B) Heatmap representing the percentage of samples of samples with high expression of a set of immune‐related biomarkers. Median gene expression values for each gene in each gene expression dataset were used as a cut‐off to designate if a sample is enriched for a specific biomarker. Different immune marker categories are represented with different colors on the left side of the heatmap. (C) Forest plot showing the odds ratios, confidence intervals, and FDR‐adjusted *P* value for immunotherapy response in each LUAD subtype when compared to all other subtypes. Odds ratio for AD7 subtype could not be calculated as 0 patients were predicted as potential responders in this subtype. A and B analysis were performed considering all gene expression datasets (*n* = 4573 LUAD samples). For C, we used pre‐computed TIDE scores for the TCGA‐LUAD dataset (*n* = 486, AD1: 86, AD2: 105, AD3: 32 AD4: 64, AD5: 106, AD6: 81, AD7: 12).

The immune infiltrate composition of each sample was quantified by applying GSVA on 21 immune cell‐specific gene signatures (Fig. 4A, Fig. S6) [25]. On the one hand, AD3 and AD5 tumors displayed higher infiltration of most immune cells, including both immune active and immunosuppressive categories. Nevertheless, there were also some distinctive features between AD3 and AD5 LUAD subtypes. For instance, AD3 subtype comprised a higher percentage of tumors with high Th2 infiltration when compared to AD5. AD4 subtype was preferentially infiltrated by innate immune cells (i.e., NK cells, neutrophils, eosinophils, and mast cells) and some specific T‐cell populations (i.e., follicular T helper cells, T effector memory cells, T effector memory cells, T gamma‐delta cells, and T helper 17). However, in AD4 subtype the presence of immunosuppressive cells (i.e., macrophages M2 and T regulatory cells) was lower than in other highly infiltrated subtypes (i.e., AD3, AD5, and AD6). AD6 tumors appeared to be more frequently enriched by T‐cell populations, both with cytotoxic and immunosuppressive roles (i.e., cytotoxic T cells, T regulatory, T helper 1, and T helper 2). AD6 subtype was also commonly infiltrated by other immunosuppressive cells (i.e., macrophages) and other innate cells (i.e., active dendritic cells and CD56dim NK cells). Finally, AD2 was, overall, the least infiltrated subtype compatible with an immune desert phenotype.

Regarding immune checkpoint and T‐cell expression markers, AD3, AD5, and AD6 were also enriched in tumors showing higher expression levels of a wide variety of the evaluated biomarkers, followed by AD4 (Fig. 4B, Fig. S7).

Finally, we also evaluated the utility of our LUAD subtypes to predict the predisposition to immunotherapy response beyond PD‐L1 and TMB biomarkers using previously calculated TIDE scores in the TCGA‐LUAD dataset [29]. We used a likelihood ratio test to compare two binomial generalized linear models (GLM) predicting immunotherapy response (i.e., yes or no). The first GLM included PD‐L1 gene expression (i.e., low and high based on median cut‐off) and TMB values as independent variables, and the second GLM was identical but also considering LUAD subtype as a predictor. Results showed that LUAD subtype further contributes to predict the probability of immunotherapy response (*P* = 0.0003). Moreover, and although not used as a stratification criterion in NSCLC in clinical trials or in the clinical practice, we also added PD‐1 expression (i.e., low and high based on median cut‐off) as a proxy of T‐cell infiltration to the model. Again, the results showed that our classification further contributes to predict the probability of immunotherapy response (*P* < 0.001). Given this outcome, for each subtype, we assessed the likelihood of immunotherapy response when compared to the tumors in any other subtypes (Fig. 4C). Tumors within AD4 subtype were found to be 2.9 times more likely to respond to immunotherapy compared to the tumors classified in any other subtype (34.4% predicted responders in AD4 [*n* = 64] vs 15.2% in other subtypes [*n* = 422]). Despite being among the most infiltrated subtypes and showing high PD‐L1 gene expression (Fig. 4A,B), only 12.5% of AD3 tumors were predicted as potential responders. Also, in correlation with its immune excluded phenotype, AD2 tumors were 80% less likely to respond to immunotherapy than other subtypes (4.76% predicted responders in AD2 [*n* = 105] vs 21.2% in other subtypes [*n* = 381]).

### 3.6 LUAD consensus subtype independent validation

**Fig. 5:**
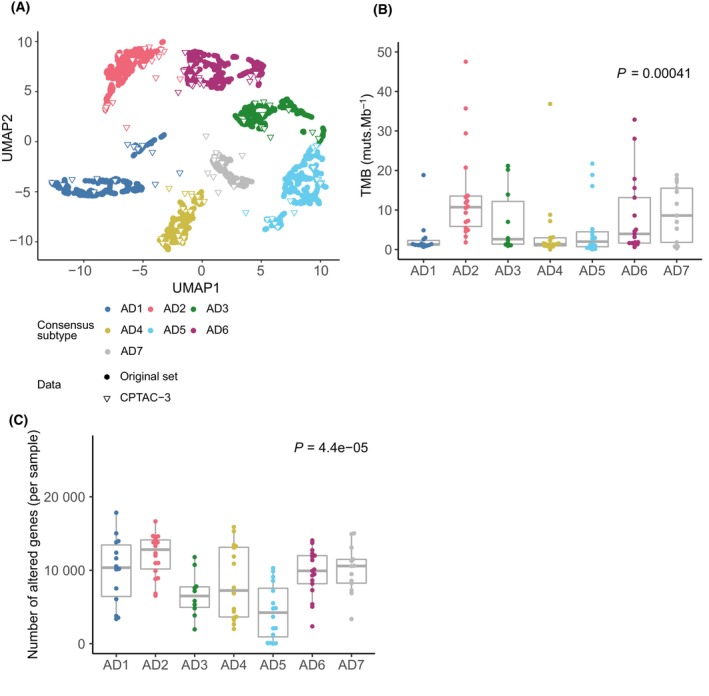
LUAD pathway transcriptional profiling‐based classification independent validation. (A) New samples from the CPTAC‐3 LUAD dataset were mapped on the previously established LUAD classification. New samples' subtype status was decided based on the most frequent label of the 51 nearest neighbors of the original classification. Colored circles represent samples used in the original set, whereas triangles represent new CPTAC‐3 validation set samples (*n* = 105). (B) Boxplot representing tumor mutational burden (TMB) values across newly classified CPTAC‐3 LUAD samples. Each dot represents the TMB value per sample (AD1:14, AD2: 18, AD3: 10, AD4: 17, AD5: 16, AD6: 17, AD7: 13). Kruskal–Wallis test was used to assess potential differences regarding TMB between LUAD subtypes. *P* value was corrected using the false discovery rate (FDR) multiple‐comparison correction method. (C) Boxplot representing copy number burden across newly classified CPTAC‐3 LUAD samples. Each dot represents the number of altered genes per sample (AD1:14, AD2: 18, AD3: 10, AD4: 17, AD5: 16, AD6: 17, AD7: 13). Kruskal–Wallis test was used to assess potential differences regarding the number of copy number altered genes between LUAD subtypes. *P* value was corrected using the false discovery rate (FDR) multiple‐comparison correction method.

We conducted an independent validation of the LUAD subtypes using CPTAC‐3 LUAD dataset [30]. The activity level of 50 molecular pathways was measured and mapped in 111 LUAD which were classified based on a *k*‐nearest‐neighbors algorithm (Fig. 5A). All seven subtypes were predicted in this independent dataset, confirming the robustness of the classification. Moreover, the pathway transcriptional footprint of each subtype is conserved between the original and the validation datasets (Fig. S8). To further prove the validity of the predictions, we explored whether the association between the LUAD subtype and copy number alterations, and TMB is conserved in the validation set (Fig. 5B,C). Notably, subtype TMB and copy number alterations rate are highly concordant between the original and validation sets, confirming that previously found associations at the genomic level are maintained (Figs 3A,C and 5B,C).

### 3.7 Analysis of drug sensitivity in *in vitro* data revealed potential therapeutic vulnerabilities for the subtypes

**Fig. 6:**
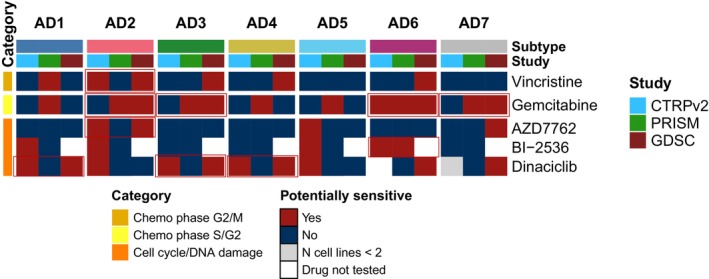
Potential treatment strategies for the LUAD subtypes based on CTRPv2, GDSC, and PRISM LUAD‐CCLs drug sensitivity data. Heatmap representing potentially suitable specific therapeutic strategies in at least two different studies within the same subtype. Mean area above the curve (AAC) sensitivity metric values were only calculated if the drug had been tested in at least two different LUAD‐CCLs within a subtype and study. Subtypes were considered as potentially sensitive to the treatment if the average AAC value for the cell lines classified within a certain group was greater than mean AAC plus 2 standard deviations for the drugs assessed in at least 2 out of the 3 evaluated pharmacogenomics studies.

Data from three large‐scale pharmacogenomics studies conducted on cancer cell lines were integrated to explore potential therapeutic vulnerabilities in LUAD. First, LUAD‐CCLs were classified according to the primary tumors' classification, and then, we assessed the impact of our classification on the response to specific compounds (Fig. 6, Fig. S9).

LUAD‐CCL subtypes were considered potentially sensitive to a specific drug whenever average AAC values were greater than the mean plus 2 standard deviations of all drugs AAC values in at least 2 out of the 3 evaluated studies. Out of 239 evaluated drugs (i.e., number of drugs tested in at least two studies), only 5 were found to be consistently effective (i.e., AAC values above threshold) in at least two studies for some of the subtypes and not the others. Overall, cells assigned to AD2 showed potential sensitivity to vincristine and gemcitabine chemotherapies, which correlates with its proliferative nature. Also, cell lines classified in AD3, AD6, and AD7 subtypes, also showing high cell cycle activity, were found to be potentially sensitive to gemcitabine treatment. Interestingly, AZD7762 CHK1 inhibitor could be potentially suitable for AD2 cell lines, which correlates with the higher genome instability described for AD2 subtype. Despite a lower cycling nature of subtype AD1 and AD4, cell lines classified within these subtypes appeared to be potentially sensitive to dinaciclib, based on these data.

## 4 Discussion

In this study, we integrated the transcriptional profiles of more than 4500 LUAD, and based on the activity levels of a set of 50 molecular pathways, we were able to identify seven LUAD molecular subtypes. Importantly, the number of samples included in this study further exceeds that of previous studies, covering the largest part of the molecular diversity of LUAD [8]. This classification was associated with survival outcomes and was correlated with relevant clinical characteristics. Besides, at the genomic level, LUAD transcriptional subtypes were associated with the presence of oncogenic driver alterations, mutational signatures, CNA burden, and DDR capacity. These results support the previously described transcriptional heterogeneity that exists within LUAD histological entity [8]. Furthermore, the integration of drug sensitivity data from three large pharmacogenomics studies unraveled potential therapeutic vulnerabilities for the subtypes. Finally, the transcriptional subtypes showed distinct patterns in terms of immune cells infiltration and immune‐related biomarkers expression and were able to predict immune response in addition to PD‐L1 gene expression and TMB.

Since early 2000s, there have been several efforts to define clinically relevant LUAD transcriptional subtypes, which resulted in various different classifications [8, 30, 35, 36]. Despite all these studies, LUAD subtypes have never been translated into the clinical setting. Reasons for this include intrinsic technical and analytical limitations, such as low overlap between the gene signatures, probably due to intrinsic technical and biological variability of individual gene expression levels. In our work, we focused on the activity levels of a set of established molecular pathways rather than in the expression of individual genes. This approach is likely to reduce the effect of the stochastic sources of variability to which multiple single‐gene measures are subjected [9]. Moreover, the method used for measuring the pathway activity (GSVA algorithm) is able to overcome batch effects compared with other deconvolution methods [10, 37]. Importantly, we were able to validate our classification framework in an independent set of samples [30]. This approach would therefore be capable to accurately classify new prospective samples into one of the specific transcriptional subtypes.

Also, we evaluated the correspondence between the widely accepted Hayes et al. mRNA subtypes and the present classification [21, 32, 33]. In summary, we found that, in most cases, our pathway transcriptional profiling‐based subtypes further stratified the ones proposed by Hayes et al., based on individual genes expression, suggesting a higher resolution of our classification to deal with the molecular heterogeneity that exists within LUAD.

The lack of association of previously described LUAD intrinsic subtypes with available therapeutic strategies prevented their clinical use. We tried to overcome this limitation by integrating drug sensitivity data from *in vitro* pharmacogenomics studies [31]. These databases have greater drug coverage compared with other available ones such as CMap, which has been used for similar purposes [35]. Although significant discrepancies can exist between drug response results obtained from cancer cell lines and clinical response in patients, we were able to identify some potential drug candidates for the different LUAD subtypes, in line with their molecular characteristics. In this way, chemotherapy alone, or combined with immunotherapy, is the cornerstone for patients with driver‐negative LUAD. However, clinical responses upon chemotherapy regimens are highly heterogeneous and underscore the need for improving patient selection [38]. In our work, we observed that AD2, AD3, AD6, and AD7 cell lines might benefit from vincristine and gemcitabine chemotherapies. Moreover, cancer cells classified as AD2 showed potential sensitivity to AZD7762 CHK1 inhibitor, which correlates with the higher genome instability seen in this subtype.

Genomic profiling is crucial in LUAD tumors to guide the most appropriate treatment based on the detection of actionable oncogenic alterations. In fact, this study does not intend to replace the current classification based on genomic profiling. However, there is a non‐negligible percentage of patients lacking tractable genomic alterations, and even patients with oncogenic drivers show heterogeneous responses to targeted therapies for reasons that remain unclear, and all patients will eventually develop treatment resistance [39]. Our results highlight the significant heterogeneity of this disease as patients with the same mutational event were found to be distributed across all subtypes, being *KRAS* mutant LUAD the most heterogeneous entity. In addition to the role of concurrent genomic alterations, differences in the activation of transcriptional pathways could explain that patients harboring identical driver alterations might have distinct clinical outcomes upon targeted therapy. Conversely, the fact that tumors with different driver alterations coexist in the same transcriptional subtype suggests that different oncogenic mutations may give rise to similar transcriptional phenotypes, which could benefit from similar combinatorial strategies. Therefore, the implementation of new methodologies beyond genomic testing, such as those based on gene expression, could help to deliver more precise and innovative treatments to patients with LUAD, specially in those patients without actionable genomic alterations or that have progressed frontline chemoimmunotherapy.

Immunotherapy alone or in combination with chemotherapy has become the standard of care for driver‐negative metastatic LUAD [3]. However, durable clinical benefit is observed only in a reduced fraction of patients (< 20%) [40]. Previous studies have shown that TMB or PD‐L1 expression cannot accurately predict long‐term benefit in all patients [41]. The improvement of patient selection and the definition of rational combinations are therefore an unmet clinical need. Transcriptomic data could provide clinically relevant information beyond individual markers. In this regard, our results showed that AD2, despite having high TMB was an immune cold subtype, and was 80% less likely to respond to immunotherapy than tumors classified in other subtypes. This result is concordant with the findings of a previous study that also identified a LUAD subtype with high TMB but no apparent immune infiltration [35]. Overall, these results underline the limitation of TMB to predict potential response to immunotherapy in LUAD [42]. We also found that although most patients classified in AD3 subtype showed higher cytotoxic T‐cell infiltration and PD‐L1 gene overexpression (*CD274*), they were also unlikely to respond to ICI therapy according to TIDE scores (12% patients were classified as responders in AD3) [43]. AD3 tumors not only co‐express a wide variety of immune checkpoint inhibitors and T‐cell exhaustion markers but also showed high infiltration of immunosuppressive cells, such as M2 macrophages and T regulatory cells, which could contribute to intrinsic resistance to immunotherapy. Thus, macrophage‐targeted therapy could be a potential solution for improving AD3 tumor response [44]. Also, AD3 shows relatively high TGF‐β signaling activity, which has previously been associated with lack of response to immunotherapy [45]. For this reason, rational combinations of ICI and immune cell‐specific targeted therapies could probably improve clinical outcomes in solid tumors. However, most clinical trials are not yet selecting patients based on the immune contexture [46, 47]. Tumors classified in AD4 subtype were 2.9 times more likely to respond to immunotherapy than tumors classified in other subtypes. These tumors showed infiltration of cytotoxic T cells and other cells involved in tumor destruction (i.e., B cells, NK cells, diverse types of T cells, etc.) and lower infiltration of immunosuppressive cells (e.g., T regulatory cells, macrophages M2, etc.), potentially constituting a less immune evasive microenvironment. Although further validation through other techniques that provide more cellular resolution (i.e., scRNA‐seq) would be needed, these results underscore the need to comprehensively characterize the immune contexture, along with conventional single biomarkers (i.e., PD‐L1 and TMB), to perform an accurate patient stratification and deliver tailored and effective treatment strategies for advanced LUAD.

Despite all the obtained results, our study has some intrinsic limitations that must be acknowledged. This is a retrospective analysis of multiple microarray and RNA‐seq gene expression studies, which rely on fresh tissue biopsies. Thus, further research is needed towards the implementation of this classification in formalin‐fixed paraffin‐embedded samples, which are routinely available in the clinical setting. For instance, we believe that with the incorporation of new profiling technologies, such as HTG EdgeSeq, which allows whole‐transcriptome gene expression profiling in FFPE samples, it will be possible to evaluate the clinical relevance of our framework using clinical samples. Moreover, although later validated in the CPTAC‐3 dataset, results regarding the association with TMB and CNA were based exclusively on the TCGA‐LUAD dataset, as the rest of the studies did not have associated WES or CNA data. Most studies included patients who were surgically resected and did not receive systemic therapy, or this information was not available. For instance, this is particularly relevant for the results regarding immunotherapy response predictions, which should be further validated in retrospective and prospective studies of patients with LUAD treated with ICI. Regarding cancer cell lines drug sensitivity results, potential drug candidates are based on *in vitro* data and do not take into consideration the interplay between cancer cells and TME. However, these models are continuously used in preclinical research for similar purposes (i.e., drug screening and hypothesis generation) and we believe that this exercise could be useful to prioritize which compounds could be tested in more advanced preclinical models (i.e., tumoroids and patient‐derived xenografts).

## 5 Conclusions

To sum up, we have presented and validated a robust and clinically relevant classification of LUAD tumors, based on the transcriptional activity levels of important cellular pathways. To our knowledge, no previous LUAD classification has been derived from such a large sample size. Despite significant challenges, we believe that the integration of transcriptomic and genomic data could improve patient stratification and may pave the way for guiding novel therapeutic approaches in patients with LUAD.

## Conflict of interest

The authors declare that they have no known competing financial interests or personal relationships that could have appeared to influence the work reported in this paper. XS participated in lectures from Roche. EN received research support from Roche, Pfizer, Merck Serono, and Bristol Myers Squibb and participated in advisory boards or lectures from Bristol Myers Squibb, Merck Serono, Merck Sharpe & Dohme, Lilly, Roche, Pfizer, Bayer, Sanofi, Takeda, Boehringer Ingelheim, Amgen, and AstraZeneca.

## Author contributions

SH‐P, XS, and EN contributed to conceptualization, formal analysis, data interpretation, writing – original draft, and writing – review and editing; DC, AA, RM, NV, RP, JB, and AM‐C contributed to data interpretation, analysis, and writing – review and editing. All authors have read and agreed to the published version of the manuscript.

### Peer review

The peer review history for this article is available at https://www.webofscience.com/api/gateway/wos/peer‐review/10.1002/1878‐0261.13550.

## Supporting information


**Fig. S1.** Flow diagram of included gene expression datasets search and filtering criteria for this study.
**Fig. S2.** Computational framework for LUAD consensus subtype definition.
**Fig. S3.** Relative activity levels (GSVA scores) of the 50 studied landmark pathways across LUAD subtypes.
**Fig. S4.** Overall survival between subtypes associated with better prognosis and worse prognosis in the analysis by individual LUAD subtype.
**Fig. S5.** Correlation between pathway profiling‐based subtypes and Wilkerson et al.'s mRNA‐based subtypes.
**Fig. S6.** Immune cell lines relative abundance across LUAD subtypes.
**Fig. S7.** Immune checkpoints expression across LUAD subtypes.
**Fig. S8.** Relative activity levels of the fifty studied pathways in each of the 111 CPTAC‐3 LUAD samples that were assigned to a consensus subtype.
**Fig. S9.** LUAD cancer cell lines (LUAD‐CCL) used for the potential treatment strategies discovery analysis.

**Table S1.** List of gene expression datasets included in this study.
**Table S2.** Correlation of dataset ids with LUAD subtypes.
**Table S3.** FDR‐adjusted p values for pairwise comparisons of TMB values between LUAD subtypes.
**Table S4.** Percentage of positive patients for each single nucleotide base substitution mutational signature across LUAD subtypes.
**Table S5.** FDR‐adjusted p values for pairwise comparisons of copy number rate values between LUAD subtypes.
**Table S6.** FDR‐adjusted p values for pairwise comparisons of copy number rate values between LUAD subtypes.

## Data Availability

Gene expression data for LUAD consensus classification were obtained from GEO and ArrayExpress public repositories. Specific information and identifiers from each dataset are available at Table S1. CCLE and GDSC LUAD cancer cell lines molecular data were obtained from https://depmap.org and https://www.cancerrxgene.org/gdsc1000/GDSC1000_WebResources/Home.html, respectively. Cellosaurus identifiers of specific cell lines used are depicted in Fig. S8. GDSC, CTRPv2, and PRISM studies drug sensitivity data were available within the pharmacogx r package [31]. LUAD CPTAC‐3 study gene expression and copy number alterations data were downloaded from the supplementary material of [30].
