# Supplementary material for: Transcriptional analysis of landmark molecular pathways in lung adenocarcinoma results in a clinically relevant classification with potential therapeutic implications
Source: Mol Oncol. 2023 Dec 21;18(2):453–70. doi: 10.1002/1878-0261.13550 (PMC10850798; doi:10.1002/1878-0261.13550)
Supplement: Supplementary file 1 — Fig. S1. Flow diagram of included gene expression datasets search and filtering criteria for this study. Fig. S2. Computational framework for LUAD consensus subtype definition. Fig. S3. Relative activity levels (GSVA scores) of the 50 studied landmark pathways across LUAD subtypes. Fig. S4. Overall survival between subtypes associated with better prognosis and worse prognosis in the analysis by individual LUAD subtype. Fig. S5. Correlation between pathway profiling‐based subtypes and Wilkerson et al.'s mRNA‐based subtypes. Fig. S6. Immune cell lines relative abundance across LUAD subtypes. Fig. S7. Immune checkpoints expression across LUAD subtypes. Fig. S8. Relative activity levels of the fifty studied pathways in each of the 111 CPTAC‐3 LUAD samples that were assigned to a consensus subtype. Fig. S9. LUAD cancer cell lines (LUAD‐CCL) used for the potential treatment strategies discovery analysis. [file MOL2-18-453-s001.docx]

SUPPLEMENTARY FIGURES

## Supplementary Fig. 1. Flow diagram of included gene expression datasets search and filtering criteria for this study.


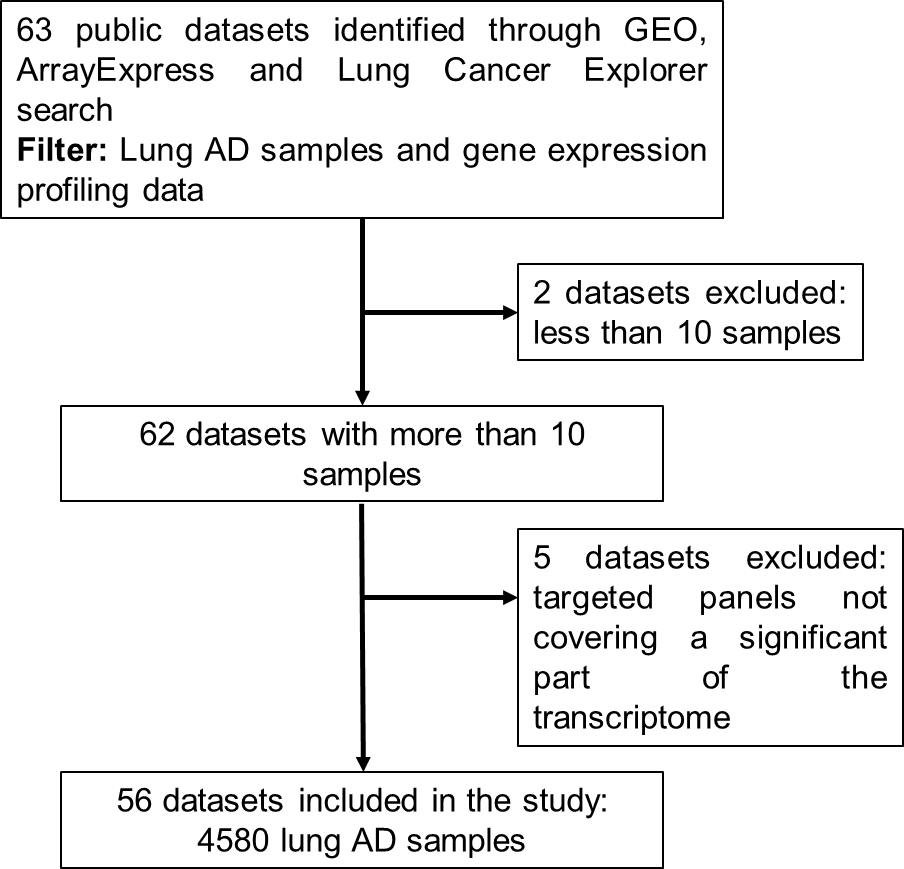


**Supplementary Fig. 2. Computational framework for LUAD consensus subtypes definition. (Next page)**

### UMAP algorithm and walktrap graph-based clustering tool with Euclidean distance were used on each of the 500 GSVA scores matrices for LUAD subpopulations identification. As a result, 500 potential classifications with different LUAD subpopulations were obtained. From here, we conducted a series of steps subsequently enumerated: 1) mean GSVA scores for each evaluated pathway were calculated for the subpopulations found within each classification; 2) UMAP and walktrap method with Euclidean distance were applied to these centroids and consensus subtypes were identified; 3) Samples assigned to centroids belonging to different consensus subtypes were allocated to the subtype to which they had been assigned the majority of times across the different classifications. If a tie existed between two consensus subtypes, the sample was eliminated from subsequent analyses. In this case seven samples were filtered out as a subtype could not be assigned. Thus, the final number of LUAD samples with an assigned consensus subtype after this classification process is 4,543.


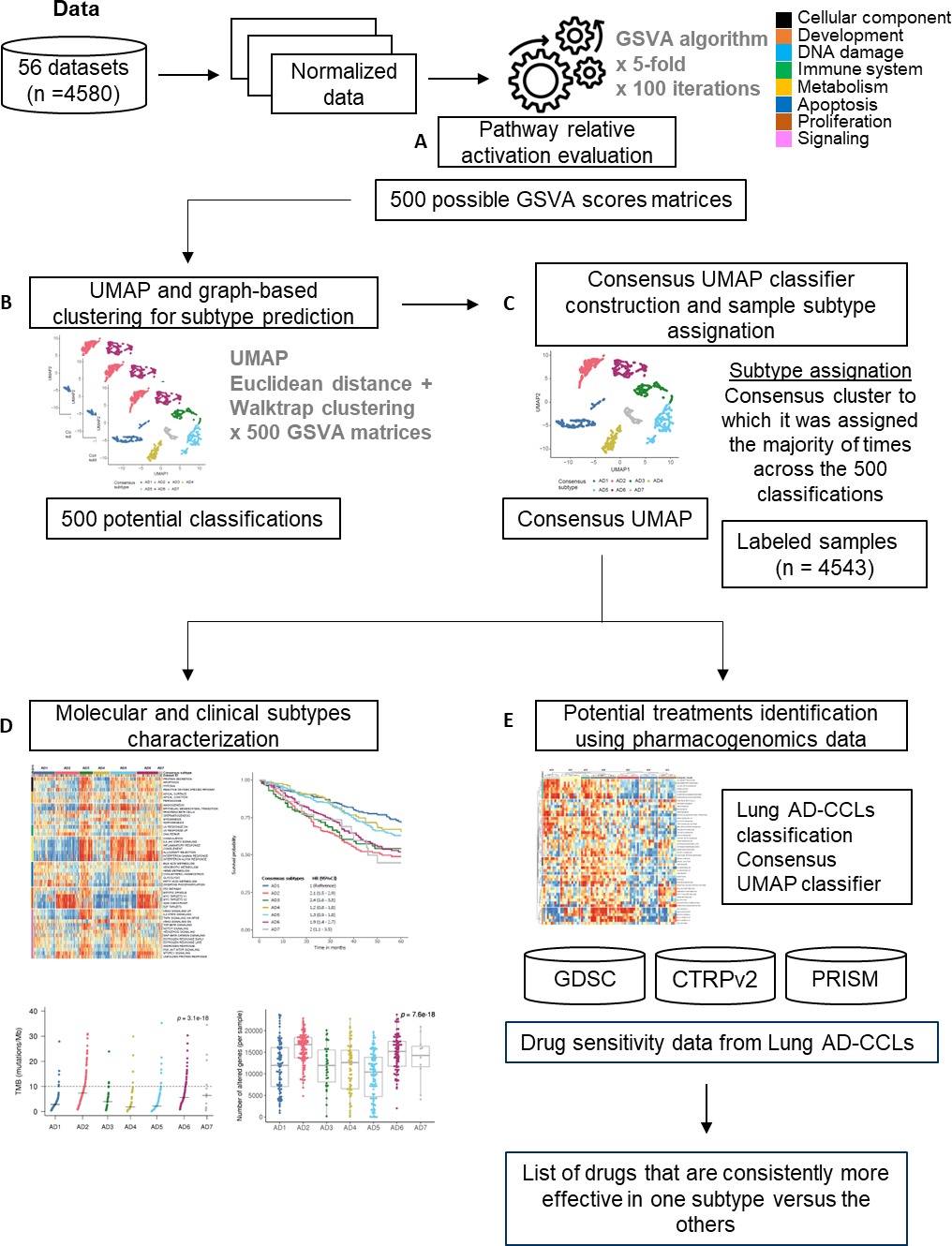


**Supplementary Fig. 3. Relative activity levels (GSVA scores) of the 50 studied landmark pathways across LUAD subtypes.** U Mann Whitney test was used to perform pairwise comparisons for each molecular pathway. *P* values were corrected using Bonferroni multiple-testing correction method. (* p < 0.05 all pairwise comparisons, ** p < 0.01 all pairwise comparisons, *** p < 0.001 all pairwise comparisons, # p < 0.05 in three pairwise comparisons, ## p < 0.01 in three pairwise comparisons, ### p < 0.001 in three pairwise comparisons. Symbols on top or bottom of the boxplots represent upregulated or downregulated pathways in a specific subtype, respectively.


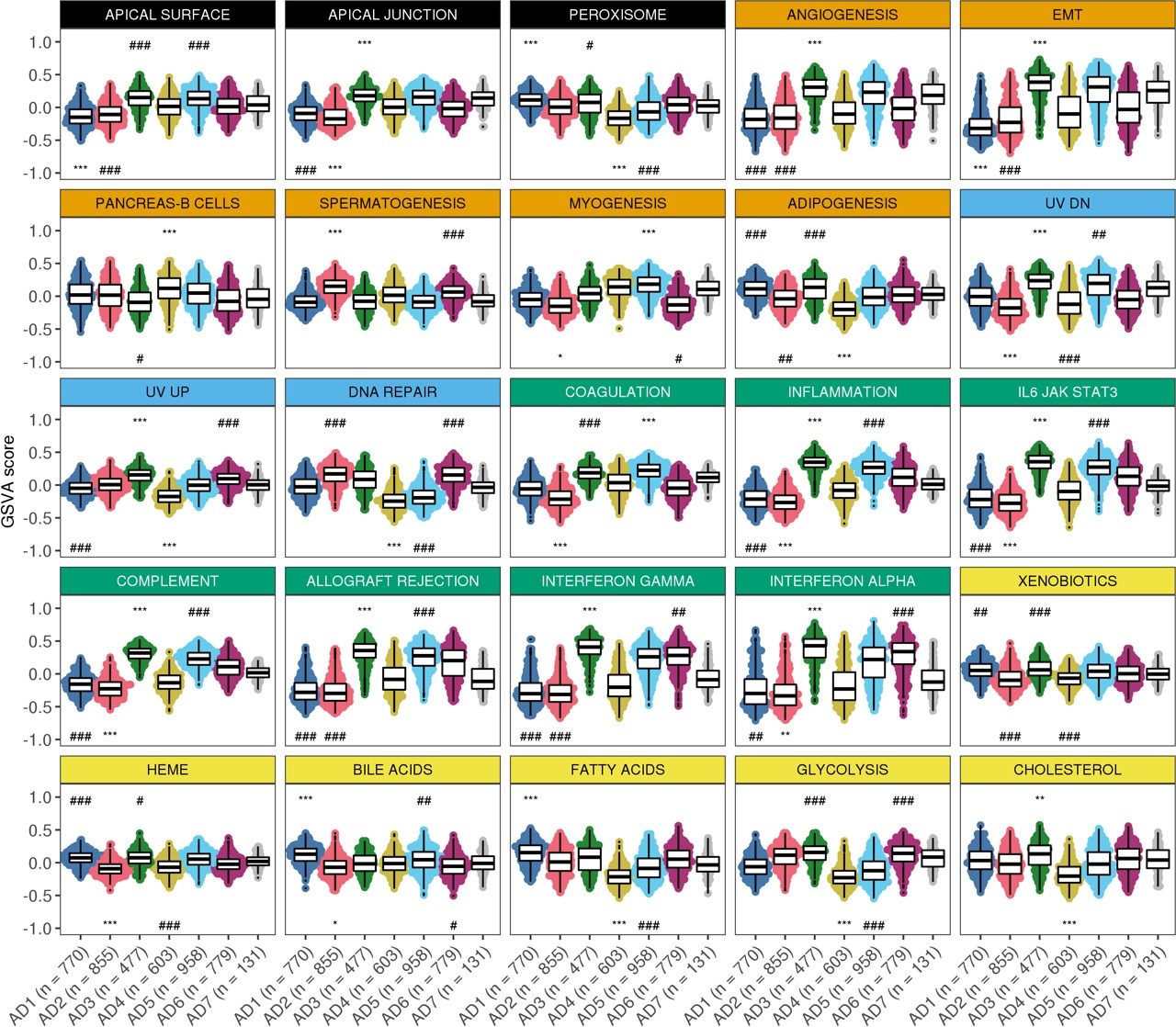


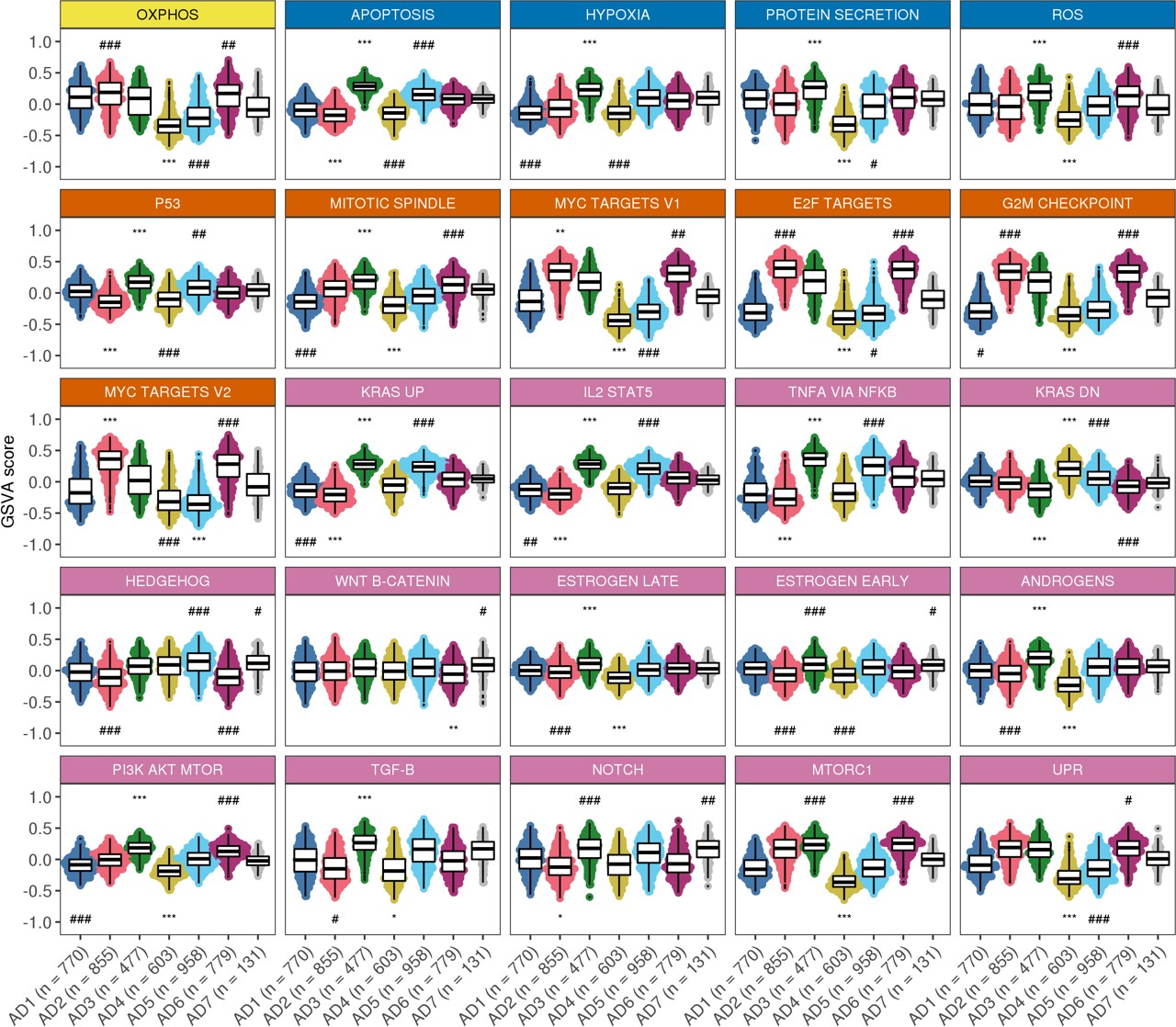


**Supplementary Fig. 4. Overall survival between subtypes associated with better prognosis and worse prognosis in the analysis by individual LUAD subtype.** Kaplan-Meier curves of pathway transcriptional profiling-based subtypes associated with better prognosis in the analysis by subtype (i.e., AD1, AD4, AD5) vs those subtypes that were associated with worse outcomes (i.e., AD2, AD3, AD6, AD7). Hazard ratios (HR) and 95% confidence intervals (95% CI) come from a Cox proportional-hazards model adjusted for age, sex, stage, smoking history, and study.


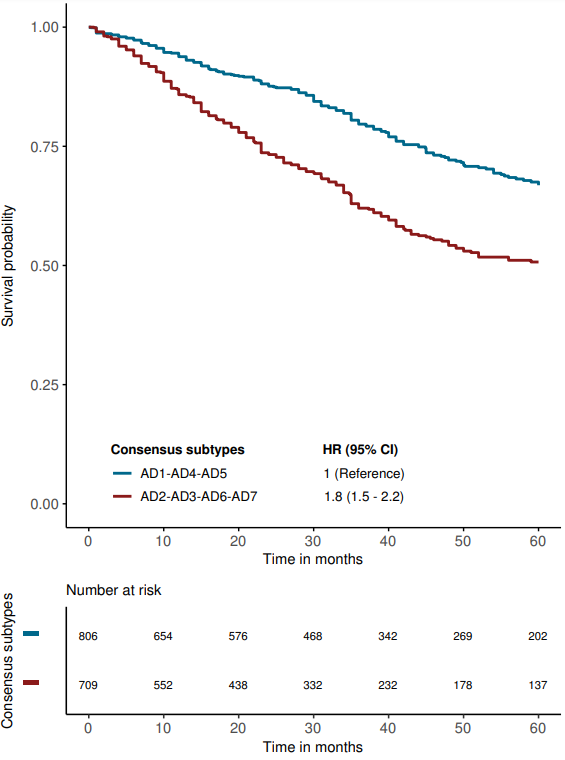


**Supplementary Fig. 5. Correlation between pathway profiling-based subtypes and Wilkerson et al.’s mRNA-based subtypes. (A)** Wilkerson et al.’s LUAD mRNA- based subtypes (i.e., secretory, basal, classical, and proliferative) were assigned to each LUAD sample using the nearest centroid predictor approach described by the original authors. The overlap degree (proportion of samples within the same category) between the two classifications is displayed in each case. **(B)** LUAD consensus pathway-based subtypes that best align with each Wilkerson et al. subtypes are displayed in each case, as well as the corresponding overlap value.


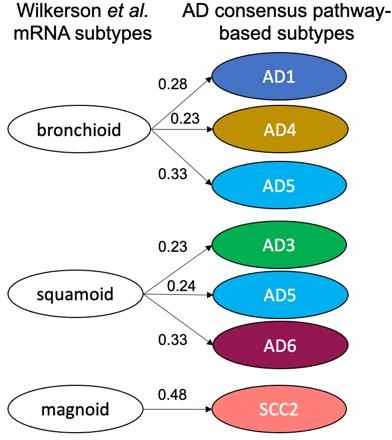
**A B**


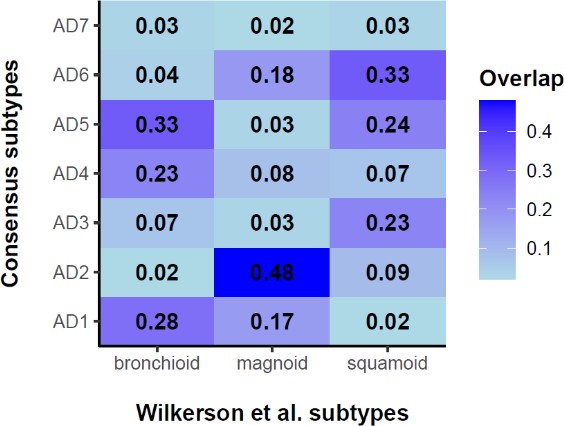


## Supplementary Fig. 6 Immune cell lines relative abundance across LUAD subtypes.

U Mann Whitney test was used to perform pairwise comparisons for each immune population. *P* values were corrected using Bonferroni multiple-testing correction method. (* p < 0.05 all pairwise comparisons, ** p < 0.01 all pairwise comparisons, *** p < 0.001 all pairwise comparisons, # p < 0.05 in three pairwise comparisons, ## p < 0.01 in three pairwise comparisons, ### p < 0.001 in three pairwise comparisons. Symbols on top or bottom of the boxplots represent upregulated or downregulated pathways in a specific subtype, respectively.


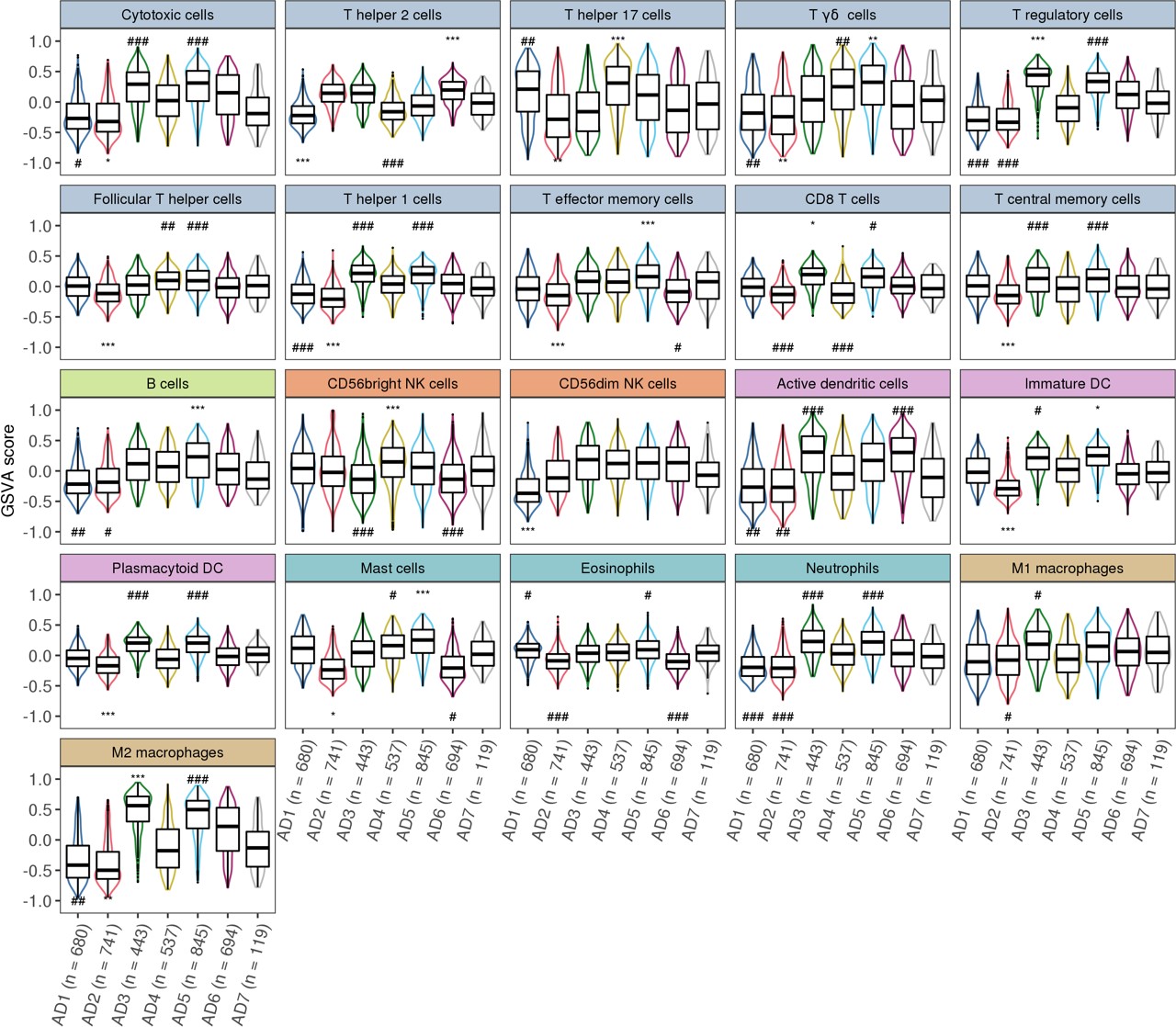


## Supplementary Fig. 7 Immune checkpoints expression. (Next page)

Expression of immune checkpoints across LUAD subtypes per dataset. Genes are plotted whenever available in each sequencing platform. Datasets are ordered in decreasing order based on the sample size.

# CD274


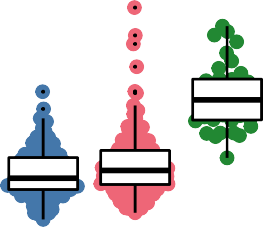

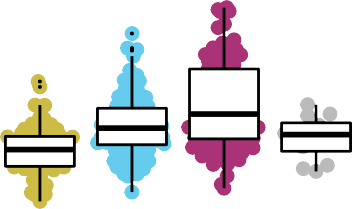


*p* = 2.4e−38

15

# PDCD1LG2

15


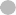

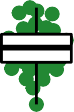

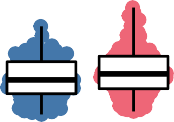

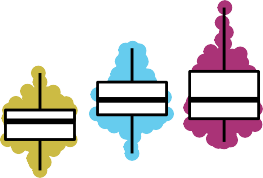


*p* = 3.7e−46

# PDCD1

15


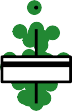

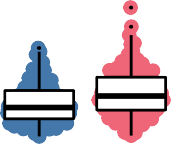

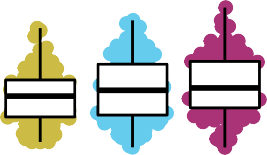


*p* = 1.6e−25

TCGA−LUAD (N = 514)

# CTLA4


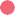

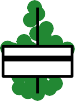

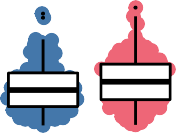

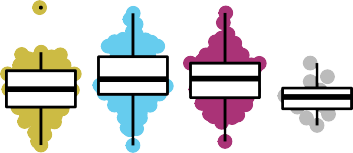


*p* = 4.8e−27

15

# HAVCR2

15

10 10 10 10 10

Expression

Expression

Expression

Expression

Expression

5 5 5 5 5

0 0 0 0 0

# LAG3


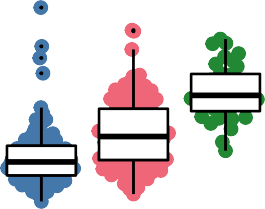

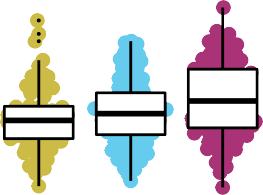


*p* = 9.9e−28

15

# TIGIT

15


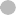

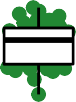

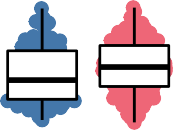

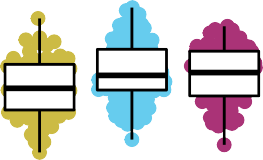


*p* = 4.2e−30

# VSIR

15


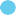

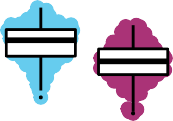

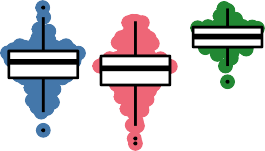

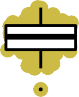


*p* = 2.2e−32

# BTLA

15


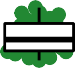

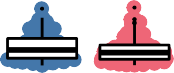

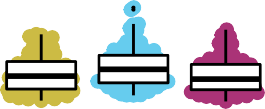


*p* = 1.9e−21

# IDO1

15

10 10 10 10 10

Expression

Expression

Expression

Expression

Expression

5 5 5 5 5

0 0 0 0 0

# CD276


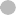

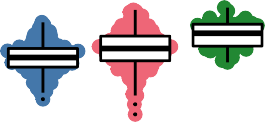

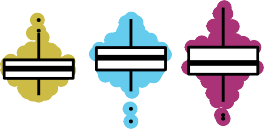


*p* = 2e−11

15

# VTCN1

15


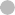

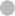

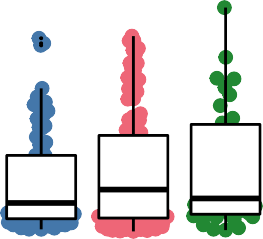

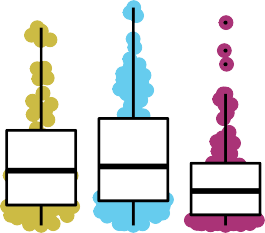


*p* = 0.0063

*GEM*

15


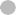

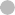

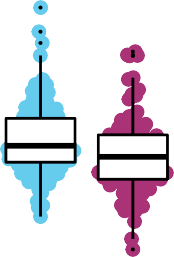

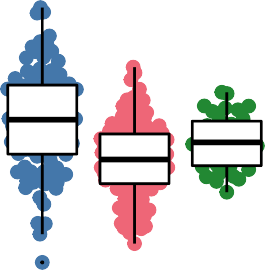

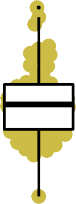


*p* = 1.2e−07


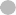

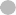

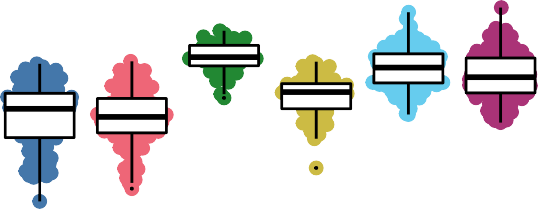


*p* = 1.7e−48


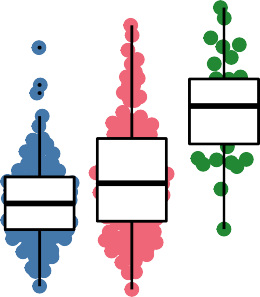

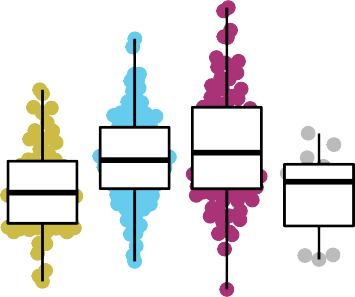


*p* = 1.3e−24

10 10 10

Expression

Expression

Expression

5 5 5

0 0 0

# PDCD1LG2


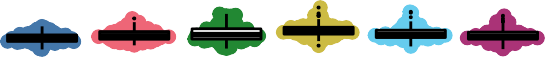


*p* = 2.6e−11

15

# PDCD1

15


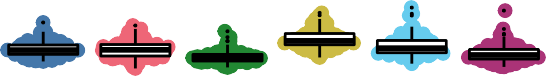


*p* = 1.3e−29

# CTLA4

15


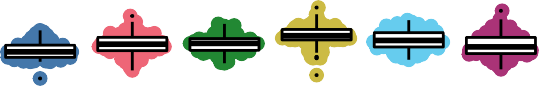


*p* = 1.6e−14

10 10 10

Expression

Expression

Expression

5 5 5

0 0 0

# LAG3


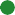

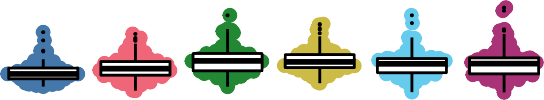


*p* = 1.9e−10

15

# IDO1

15

10 10

Expression

Expression

5 5

0 0

# VTCN1


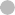

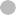

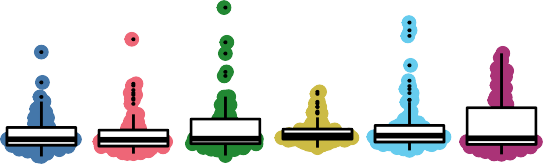


*p* = 0.018

15

*GEM*

15


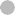

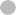

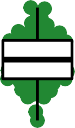

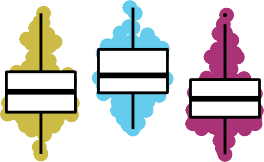

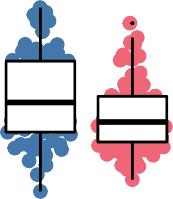


*p* = 8e−07


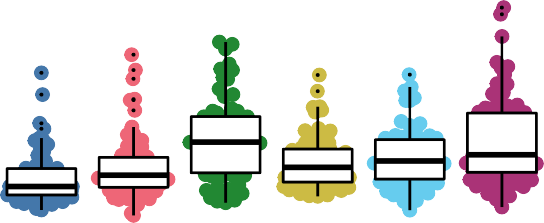


*p* = 1.3e−11

10 10

Expression

Expression

5 5

0 0

# CD274


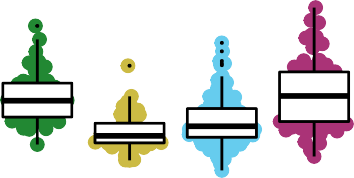

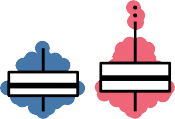

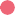

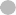


*p* = 3.3e−31

15

# PDCD1LG2

15


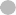

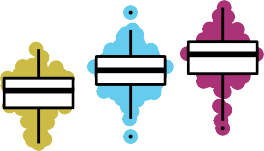

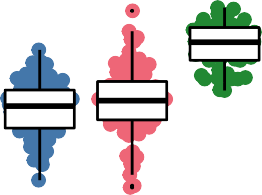

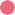


*p* = 8.8e−40

10 10

Expression

Expression

5 5

0 0

# TIGIT


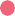

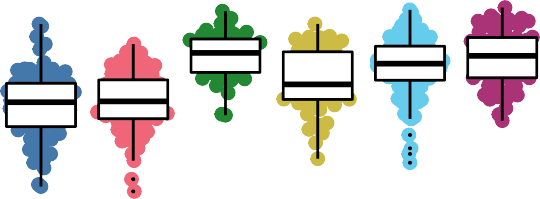

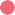


*p* = 7.4e−28

15

# VSIR

15


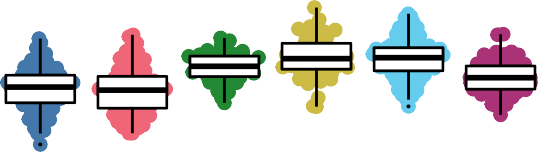


*p* = 8.3e−28

# BTLA

15


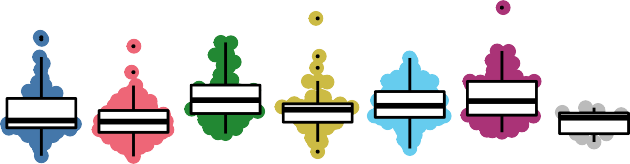


*p* = 2.2e−13

10 10 10

Expression

Expression

Expression

5 5 5

0 0 0

# CD274


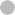

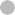

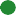

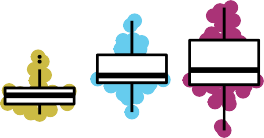

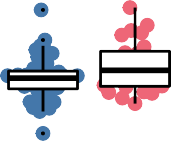


*p* = 1.3e−11

15

# PDCD1LG2

15


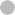

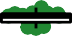

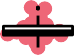

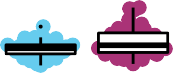

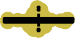

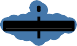


*p* = 3.1e−11

# PDCD1

15


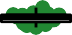

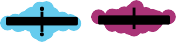

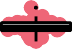

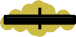

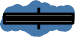


*p* = 0.13

# CTLA4

15


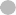

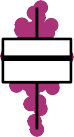

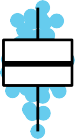

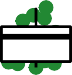

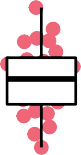

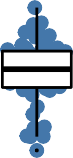


*p* = 2.2e−12

# HAVCR2

15

10 10 10 10 10

Expression

Expression

Expression

Expression

Expression

5 5 5 5 5

0 0 0 0 0

# LAG3

*p* = 5.7e−13

15

# TIGIT

15

*p* = 5.7e−13

# VSIR

15

*p* = 2.3e−11

# BTLA

15

*p* = 8.9e−10

# IDO1

15

10 10 10 10 10

Expression

Expression

Expression

Expression

Expression

5 5 5 5 5

0 0 0 0 0

# CD276

*p* = 1.8e−08

15

# VTCN1

15

*p* = 0.25

*GEM*

15

*p* = 6.3e−08

*p* = 5.9e−16

*p* = 4.2e−13

10 10 10

Expression

Expression

Expression

5 5 5

0 0 0

# CD274

*p* = 1.2e−05

15

# PDCD1LG2

15

*p* = 0.0014

# PDCD1

15

*p* = 3.3e−11

# CTLA4

15

*p* = 1.2e−12

# HAVCR2

15

10 10 10 10 10

Expression

Expression

Expression

Expression

Expression

5 5 5 5 5

0 0 0 0 0

# LAG3

*p* = 7.6e−10

15

# TIGIT

15

*p* = 1.4e−10

# VSIR

15

*p* = 3.3e−11

# BTLA

15

*p* = 9.2e−08

# IDO1

15

10 10 10 10 10

Expression

Expression

Expression

Expression

Expression

5 5 5 5 5

0 0 0 0 0

# CD276

*p* = 0.0015

15

# VTCN1

15

*p* = 0.39

*GEM*

15

*p* = 1.2e−05

*p* = 9.9e−17

*p* = 2.1e−07

10 10 10

Expression

Expression

Expression

5 5 5

0 0 0

# CD274

*p* = 7.3e−07

15

# CTLA4

15

*p* = 0.36

10 10

Expression

Expression

5 5

0 0

# VSIR

*p* = 0.091

15

# BTLA

15

*p* = 0.0077

# IDO1

15

10 10 10

Expression

Expression

Expression

5 5 5

0 0 0

# CD276

*p* = 0.0015

15

# VTCN1

15

*p* = 0.026

*GEM*

15

*p* = 0.00051

*p* = 0.00025

10 10 10

Expression

Expression

Expression

5 5 5

0 0 0

# CD274

*p* = 3.3e−06

15

# PDCD1LG2

15

*p* = 0.00046

# PDCD1

15

*p* = 2.7e−08

# CTLA4

15

*p* = 8.5e−09

# HAVCR2

15

10 10 10 10 10

Expression

Expression

Expression

Expression

Expression

5 5 5 5 5

0 0 0 0 0

# LAG3

*p* = 3.3e−06

15

# TIGIT

15

*p* = 8.5e−09

# VSIR

15

*p* = 2.7e−08

# BTLA

15

*p* = 6.4e−06

# IDO1

15

10 10 10 10 10

Expression

Expression

Expression

Expression

Expression

5 5 5 5 5

0 0 0 0 0

# CD276

*p* = 0.0016

15

# VTCN1

15

*p* = 0.28

*GEM*

15

*p* = 0.024

*p* = 1.1e−11

*p* = 7.9e−06

10 10 10

Expression

Expression

Expression

5 5 5

0 0 0

# CD274

*p* = 2.8e−08

15

# PDCD1LG2

15

*p* = 0.00034

# PDCD1

15

*p* = 0.00049

# CTLA4

15

*p* = 0.00018

# HAVCR2

15

10 10 10 10 10

Expression

Expression

Expression

Expression

Expression

5 5 5 5 5

0 0 0 0 0

# LAG3

*p* = 0.00031

15

# TIGIT

15

*p* = 0.00034

# VSIR

15

*p* = 0.00011

# BTLA

15

*p* = 0.00049

# IDO1

15

10 10 10 10 10

Expression

Expression

Expression

Expression

Expression

5 5 5 5 5

0 0 0 0 0

# CD276

*p* = 0.028

15

# VTCN1

15

*p* = 0.11

*GEM*

15

*p* = 0.00052

*p* = 5.2e−08

*p* = 5e−04

10 10 10

Expression

Expression

Expression

5 5 5

0 0 0

# CD274

*p* = 1.3e−08

15

# PDCD1LG2

15

*p* = 1.9e−06

# PDCD1

15

*p* = 7.2e−08

# CTLA4

15

*p* = 3.7e−07

# HAVCR2

15

10 10 10 10 10

Expression

Expression

Expression

Expression

Expression

5 5 5 5 5

0 0 0 0 0

# LAG3

*p* = 1.9e−08

15

# TIGIT

15

*p* = 6.1e−08

# VSIR

15

*p* = 1.3e−08

# BTLA

15

*p* = 3.9e−06

# IDO1

15

10 10 10 10 10

Expression

Expression

Expression

Expression

Expression

5 5 5 5 5

0 0 0 0 0

# CD276

*p* = 0.093

15

# VTCN1

15

*p* = 0.49

*GEM*

15

*p* = 0.0012

*p* = 8.2e−12

*p* = 1.5e−05

10 10 10

Expression

Expression

Expression

5 5 5

0 0 0

# CD274

*p* = 6.6e−07

15

# PDCD1LG2

15

*p* = 7.4e−07

# PDCD1

15

*p* = 5.8e−09

# CTLA4

15

*p* = 1.1e−06

# HAVCR2

15

10 10 10 10 10

Expression

Expression

Expression

Expression

Expression

5 5 5 5 5

0 0 0 0 0

# LAG3

*p* = 2.1e−10

15

# TIGIT

15

*p* = 1.1e−08

# VSIR

15

*p* = 1.7e−09

# BTLA

15

*p* = 2.3e−05

# IDO1

15

10 10 10 10 10

Expression

Expression

Expression

Expression

Expression

5 5 5 5 5

0 0 0 0 0

# CD276

*p* = 0.039

15

# VTCN1

15

*p* = 0.16

*GEM*

15

*p* = 0.0012

*p* = 4.2e−08

*p* = 2.2e−05

10 10 10

Expression

Expression

Expression

5 5 5

0 0 0

# CD274

*p* = 2.4e−08

15

# PDCD1LG2

15

*p* = 0.018

# PDCD1

15

*p* = 0.7

# CTLA4

15

*p* = 0.00023

# HAVCR2

15

10 10 10 10 10

Expression

Expression

Expression

Expression

Expression

5 5 5 5 5

0 0 0 0 0

# LAG3

*p* = 0.0042

15

# TIGIT

15

*p* = 1e−05

# VSIR

15

*p* = 1.2e−05

# BTLA

15

*p* = 0.0014

# IDO1

15

10 10 10 10 10

Expression

Expression

Expression

Expression

Expression

5 5 5 5 5

0 0 0 0 0

# CD276

*p* = 0.19

15

# VTCN1

15

*p* = 0.34

*GEM*

15

*p* = 0.12

*p* = 1.7e−09

*p* = 0.0011

10 10 10

Expression

Expression

Expression

5 5 5

0 0 0

# CD274

*p* = 0.00014

15

# PDCD1LG2

15

*p* = 0.00037

# PDCD1

15

*p* = 0.88

# CTLA4

15

*p* = 0.00064

# HAVCR2

15

10 10 10 10 10

Expression

Expression

Expression

Expression

Expression

5 5 5 5 5

0 0 0 0 0

# LAG3

*p* = 0.13

15

# TIGIT

15

*p* = 0.00064

# VSIR

15

*p* = 1.5e−05

# BTLA

15

*p* = 0.0072

# IDO1

15

10 10 10 10 10

Expression

Expression

Expression

Expression

Expression

5 5 5 5 5

0 0 0 0 0

# CD276

*p* = 0.00037

15

# VTCN1

15

*p* = 0.87

*GEM*

15

*p* = 0.057

*p* = 2.1e−06

*p* = 0.12

10 10 10

Expression

Expression

Expression

5 5 5

0 0 0

# PDCD1LG2

*p* = 0.00036

15

# PDCD1

15

*p* = 0.022

# CTLA4

15

*p* = 0.022

10 10 10

Expression

Expression

Expression

5 5 5

0 0 0

# LAG3

*p* = 0.36

15

# IDO1

15

10 10

Expression

Expression

5 5

0 0

# VTCN1

*p* = 0.89

15

*GEM*

15

*p* = 0.0088

*p* = 0.022

10 10

Expression

Expression

5 5

0 0

# CD274

*p* = 0.00049

15

# PDCD1LG2

15

*p* = 0.00039

# PDCD1

15

*p* = 1e−04

# CTLA4

15

*p* = 8.4e−06

# HAVCR2

15

10 10 10 10 10

Expression

Expression

Expression

Expression

Expression

5 5 5 5 5

0 0 0 0 0

# LAG3

*p* = 0.00049

15

# TIGIT

15

*p* = 0.15

# BTLA

15

*p* = 0.11

# IDO1

15

10 10 10 10

Expression

Expression

Expression

Expression

5 5 5 5

0 0 0 0

# CD276

*p* = 0.11

15

# VTCN1

15

*p* = 0.37

*GEM*

15

*p* = 0.17

*p* = 9.3e−06

*p* = 0.0014

10 10 10

Expression

Expression

Expression

5 5 5

0 0 0

# CD274

*p* = 0.00066

15

# PDCD1LG2

15

*p* = 0.0021

# PDCD1

15

*p* = 0.15

# CTLA4

15

*p* = 0.011

# HAVCR2

15

10 10 10 10 10

Expression

Expression

Expression

Expression

Expression

5 5 5 5 5

0 0 0 0 0

# LAG3

*p* = 0.0056

15

# TIGIT

15

*p* = 0.00066

# VSIR

15

*p* = 3.7e−06

# BTLA

15

*p* = 0.0056

# IDO1

15

10 10 10 10 10

Expression

Expression

Expression

Expression

Expression

5 5 5 5 5

0 0 0 0 0

# CD276

*p* = 0.0058

15

# VTCN1

15

*p* = 0.36

*GEM*

15

*p* = 0.043

*p* = 3.7e−06

*p* = 0.021

10 10 10

Expression

Expression

Expression

5 5 5

0 0 0

# CD274

*p* = 3.7e−07

15

# PDCD1LG2

15

*p* = 3.7e−07

# PDCD1

15

*p* = 0.027

# CTLA4

15

*p* = 0.0011

# HAVCR2

15

10 10 10 10 10

Expression

Expression

Expression

Expression

Expression

5 5 5 5 5

0 0 0 0 0

# LAG3

*p* = 0.012

15

# TIGIT

15

*p* = 0.00025

# VSIR

15

*p* = 3.2e−06

# BTLA

15

*p* = 0.0095

# IDO1

15

10 10 10 10 10

Expression

Expression

Expression

Expression

Expression

5 5 5 5 5

0 0 0 0 0

# CD276

*p* = 0.41

15

# VTCN1

15

*p* = 0.41

*GEM*

15

*p* = 0.14

*p* = 3.7e−07

*p* = 0.0021

10 10 10

Expression

Expression

Expression

5 5 5

0 0 0

# CD274

*p* = 0.0012

15

# PDCD1LG2

15

*p* = 0.22

# PDCD1

15

*p* = 0.00013

# CTLA4

15

*p* = 1.4e−05

# HAVCR2

15

10 10 10 10 10

Expression

Expression

Expression

Expression

Expression

5 5 5 5 5

0 0 0 0 0

# LAG3

*p* = 2e−04

15

# TIGIT

15

*p* = 7.5e−05

# VSIR

15

*p* = 3e−06

# BTLA

15

*p* = 0.00025

# IDO1

15

10 10 10 10 10

Expression

Expression

Expression

Expression

Expression

5 5 5 5 5

0 0 0 0 0

# CD276

*p* = 0.78

15

# VTCN1

15

*p* = 0.88

*GEM*

15

*p* = 0.67

*p* = 3e−06

*p* = 0.003

10 10 10

Expression

Expression

Expression

5 5 5

0 0 0

# CD274

*p* = 0.00019

15

# PDCD1LG2

15

*p* = 0.00019

# PDCD1

15

*p* = 1.9e−05

# CTLA4

15

*p* = 0.00029

# HAVCR2

15

10 10 10 10 10

Expression

Expression

Expression

Expression

Expression

5 5 5 5 5

0 0 0 0 0

# LAG3

*p* = 1.4e−05

15

# TIGIT

15

*p* = 0.00015

# VSIR

15

*p* = 0.00022

# BTLA

15

*p* = 0.00029

# IDO1

15

10 10 10 10 10

Expression

Expression

Expression

Expression

Expression

5 5 5 5 5

0 0 0 0 0

# CD276

*p* = 0.23

15

# VTCN1

15

*p* = 0.1

*GEM*

15

*p* = 0.00034

*p* = 0.00043

*p* = 0.00028

10 10 10

Expression

Expression

Expression

5 5 5

0 0 0

# CD274

*p* = 2.4e−05

15

# PDCD1LG2

15

*p* = 0.00035

# PDCD1

15

*p* = 0.6

# CTLA4

15

*p* = 0.0016

# HAVCR2

15

10 10 10 10 10

Expression

Expression

Expression

Expression

Expression

5 5 5 5 5

0 0 0 0 0

# LAG3

*p* = 0.0067

15

# TIGIT

15

*p* = 0.016

# VSIR

15

*p* = 5.1e−05

# BTLA

15

*p* = 0.05

# IDO1

15

10 10 10 10 10

Expression

Expression

Expression

Expression

Expression

5 5 5 5 5

0 0 0 0 0

# CD276

*p* = 0.77

15

# VTCN1

15

*p* = 0.91

*GEM*

15

*p* = 0.026

*p* = 1.6e−05

*p* = 0.0032

10 10 10

Expression

Expression

Expression

5 5 5

0 0 0

# CD274

*p* = 8e−05

15

# PDCD1LG2

15

*p* = 0.33

# PDCD1

15

*p* = 0.019

# CTLA4

15

*p* = 0.02

# HAVCR2

15

10 10 10 10 10

Expression

Expression

Expression

Expression

Expression

5 5 5 5 5

0 0 0 0 0

# LAG3

*p* = 0.017

15

# TIGIT

15

*p* = 0.0013

# VSIR

15

*p* = 9.2e−07

# BTLA

15

*p* = 5e−04

# IDO1

15

10 10 10 10 10

Expression

Expression

Expression

Expression

Expression

5 5 5 5 5

0 0 0 0 0

# CD276

*p* = 0.079

15

# VTCN1

15

*p* = 0.23

*GEM*

15

*p* = 0.0013

*p* = 1.3e−05

*p* = 5e−04

10 10 10

Expression

Expression

Expression

5 5 5

0 0 0

# CD274

*p* = 0.0013

15

# PDCD1LG2

15

*p* = 0.014

# PDCD1

15

*p* = 0.33

# CTLA4

15

*p* = 5.6e−05

# HAVCR2

15

10 10 10 10 10

Expression

Expression

Expression

Expression

Expression

5 5 5 5 5

0 0 0 0 0

# LAG3

*p* = 0.014

15

# TIGIT

15

*p* = 3.6e−05

# VSIR

15

*p* = 7.9e−06

# BTLA

15

*p* = 0.0049

# IDO1

15

10 10 10 10 10

Expression

Expression

Expression

Expression

Expression

5 5 5 5 5

0 0 0 0 0

# CD276

*p* = 0.33

15

# VTCN1

15

*p* = 0.57

*GEM*

15

*p* = 0.011

*p* = 4.4e−06

*p* = 0.0033

10 10 10

Expression

Expression

Expression

5 5 5

0 0 0

# CD274

*p* = 6.6e−05

15

# PDCD1LG2

15

*p* = 0.0014

# PDCD1

15

*p* = 0.084

# CTLA4

15

*p* = 0.0014

# HAVCR2

15

10 10 10 10 10

Expression

Expression

Expression

Expression

Expression

5 5 5 5 5

0 0 0 0 0

# LAG3

*p* = 0.0013

15

# TIGIT

15

*p* = 0.00016

# VSIR

15

*p* = 0.041

# BTLA

15

*p* = 0.0014

# IDO1

15

10 10 10 10 10

Expression

Expression

Expression

Expression

Expression

5 5 5 5 5

0 0 0 0 0

# CD276

*p* = 0.0017

15

# VTCN1

15

*p* = 0.075

*GEM*

15

*p* = 0.00016

*p* = 0.00025

*p* = 0.00056

10 10 10

Expression

Expression

Expression

5 5 5

0 0 0

# CD274

*p* = 0.00045

15

# PDCD1LG2

15

*p* = 0.023

# PDCD1

15

*p* = 0.014

# CTLA4

15

*p* = 0.0035

# HAVCR2

15

10 10 10 10 10

Expression

Expression

Expression

Expression

Expression

5 5 5 5 5

0 0 0 0 0

# LAG3

*p* = 0.032

15

# TIGIT

15

*p* = 0.014

# VSIR

15

*p* = 0.014

# BTLA

15

*p* = 0.0072

# IDO1

15

10 10 10 10 10

Expression

Expression

Expression

Expression

Expression

5 5 5 5 5

0 0 0 0 0

# CD276

*p* = 0.12

15

# VTCN1

15

*p* = 0.6

*GEM*

15

*p* = 0.032

*p* = 0.00083

*p* = 0.012

10 10 10

Expression

Expression

Expression

5 5 5

0 0 0

# CD274

*p* = 0.2

15

# PDCD1LG2

15

*p* = 0.65

# PDCD1

15

*p* = 0.2

# CTLA4

15

*p* = 0.15

# HAVCR2

15

10 10 10 10 10

Expression

Expression

Expression

Expression

Expression

5 5 5 5 5

0 0 0 0 0

# LAG3

*p* = 0.15

15

# TIGIT

15

*p* = 0.15

# VSIR

15

*p* = 0.15

# BTLA

15

*p* = 0.15

# IDO1

15

10 10 10 10 10

Expression

Expression

Expression

Expression

Expression

5 5 5 5 5

0 0 0 0 0

# CD276

*p* = 0.15

15

# VTCN1

15

*p* = 0.65

*GEM*

15

*p* = 0.15

*p* = 0.15

*p* = 0.15

10 10 10

Expression

Expression

Expression

5 5 5

0 0 0

# CD274

*p* = 0.12

15

# PDCD1LG2

15

*p* = 0.48

# PDCD1

15

*p* = 0.023

# CTLA4

15

*p* = 0.0055

# HAVCR2

15

10 10 10 10 10

Expression

Expression

Expression

Expression

Expression

5 5 5 5 5

0 0 0 0 0

# LAG3

*p* = 0.0055

15

# TIGIT

15

*p* = 0.12

# VSIR

15

*p* = 0.0055

# BTLA

15

*p* = 0.023

# IDO1

15

10 10 10 10 10

Expression

Expression

Expression

Expression

Expression

5 5 5 5 5

0 0 0 0 0

# CD276

*p* = 0.12

15

# VTCN1

15

*p* = 0.81

*GEM*

15

*p* = 0.81

*p* = 0.00012

*p* = 0.00012

10 10 10

Expression

Expression

Expression

5 5 5

0 0 0

# PDCD1LG2

*p* = 0.93

15

# PDCD1

15

*p* = 0.34

# CTLA4

15

*p* = 0.93

10 10 10

Expression

Expression

Expression

5 5 5

0 0 0

# LAG3

*p* = 0.93

15

# IDO1

15

10 10

Expression

Expression

5 5

0 0

# VTCN1

*p* = 0.93

15

*GEM*

15

*p* = 0.34

*p* = 0.3

10 10

Expression

Expression

5 5

0 0

# CD274

*p* = 0.21

15

# PDCD1LG2

15

*p* = 0.017

# PDCD1

15

*p* = 0.007

# CTLA4

15

*p* = 0.026

# HAVCR2

15

10 10 10 10 10

Expression

Expression

Expression

Expression

Expression

5 5 5 5 5

0 0 0 0 0

# LAG3

*p* = 0.00095

15

# TIGIT

15

*p* = 0.15

# VSIR

15

*p* = 0.00095

# BTLA

15

*p* = 0.15

# IDO1

15

10 10 10 10 10

Expression

Expression

Expression

Expression

Expression

5 5 5 5 5

0 0 0 0 0

# CD276

*p* = 0.007

15

# VTCN1

15

*p* = 0.0011

*GEM*

15

*p* = 0.0018

*p* = 0.035

*p* = 0.3

10 10 10

Expression

Expression

Expression

5 5 5

0 0 0

# CD274

*p* = 0.00024

15

# PDCD1LG2

15

*p* = 5e−04

# PDCD1

15

*p* = 0.00083

# CTLA4

15

*p* = 0.011

# HAVCR2

15

10 10 10 10 10

Expression

Expression

Expression

Expression

Expression

5 5 5 5 5

0 0 0 0 0

# LAG3

*p* = 0.00083

15

# TIGIT

15

*p* = 5e−04

# VSIR

15

*p* = 0.00083

# BTLA

15

*p* = 0.00064

# IDO1

15

10 10 10 10 10

Expression

Expression

Expression

Expression

Expression

5 5 5 5 5

0 0 0 0 0

# CD276

*p* = 0.46

15

# VTCN1

15

*p* = 0.87

*GEM*

15

*p* = 0.047

*p* = 0.00019

*p* = 0.0081

10 10 10

Expression

Expression

Expression

5 5 5

0 0 0

# CD274

*p* = 0.027

15

# PDCD1LG2

15

*p* = 0.33

# PDCD1

15

*p* = 0.11

# CTLA4

15

*p* = 0.35

# HAVCR2

15

10 10 10 10 10

Expression

Expression

Expression

Expression

Expression

5 5 5 5 5

0 0 0 0 0

# LAG3

*p* = 0.11

15

# TIGIT

15

*p* = 0.025

# VSIR

15

*p* = 0.0053

# BTLA

15

*p* = 0.18

# IDO1

15

10 10 10 10 10

Expression

Expression

Expression

Expression

Expression

5 5 5 5 5

0 0 0 0 0

# CD276

*p* = 0.021

15

# VTCN1

15

*p* = 0.94

*GEM*

15

*p* = 0.78

*p* = 2e−04

*p* = 0.01

10 10 10

Expression

Expression

Expression

5 5 5

0 0 0

# CD274

*p* = 0.0044

15

# PDCD1LG2

15

*p* = 0.044

# PDCD1

15

*p* = 0.43

# CTLA4

15

*p* = 0.039

# HAVCR2

15

10 10 10 10 10

Expression

Expression

Expression

Expression

Expression

5 5 5 5 5

0 0 0 0 0

# LAG3

*p* = 0.039

15

# TIGIT

15

*p* = 0.049

# VSIR

15

*p* = 0.0057

# BTLA

15

*p* = 0.049

# IDO1

15

10 10 10 10 10

Expression

Expression

Expression

Expression

Expression

5 5 5 5 5

0 0 0 0 0

# CD276

*p* = 0.43

15

# VTCN1

15

*p* = 0.47

*GEM*

15

*p* = 0.43

*p* = 0.0029

*p* = 0.044

10 10 10

Expression

Expression

Expression

5 5 5

0 0 0

15 15 15

*p* = 0.85

*p* = 0.56

*p* = 0.85

10 10 10

Expression

Expression

Expression

5 5 5

0 0 0

# LAG3

*p* = 0.85

15

# IDO1

15

10 10

Expression

Expression

5 5

0 0

# VTCN1

*p* = 0.85

15

*GEM*

15

*p* = 0.055

*p* = 0.85

10 10

Expression

Expression

5 5

0 0

# PDCD1LG2

*p* = 0.98

15

# PDCD1

15

*p* = 0.22

# CTLA4

15

*p* = 0.98

10 10 10

Expression

Expression

Expression

5 5 5

0 0 0

# LAG3

*p* = 0.59

15

# IDO1

15

10 10

Expression

Expression

5 5

0 0

# VTCN1

*p* = 0.59

15

*GEM*

15

*p* = 0.02

*p* = 0.59

10 10

Expression

Expression

5 5

0 0

# CD274

*p* = 0.022

15

# PDCD1LG2

15

*p* = 0.17

# PDCD1

15

*p* = 0.057

# CTLA4

15

*p* = 0.13

# HAVCR2

15

10 10 10 10 10

Expression

Expression

Expression

Expression

Expression

5 5 5 5 5

0 0 0 0 0

# LAG3

*p* = 0.81

15

# TIGIT

15

*p* = 0.12

# VSIR

15

*p* = 0.022

# BTLA

15

*p* = 0.094

# IDO1

15

10 10 10 10 10

Expression

Expression

Expression

Expression

Expression

5 5 5 5 5

0 0 0 0 0

# CD276

*p* = 0.89

15

# VTCN1

15

*p* = 0.89

*GEM*

15

*p* = 0.12

*p* = 0.093

*p* = 0.17

10 10 10

Expression

Expression

Expression

5 5 5

0 0 0

# CD274

*p* = 0.18

15

# PDCD1LG2

15

*p* = 0.39

# PDCD1

15

*p* = 0.22

# CTLA4

15

*p* = 0.35

# HAVCR2

15

10 10 10 10 10

Expression

Expression

Expression

Expression

Expression

5 5 5 5 5

0 0 0 0 0

# LAG3

*p* = 0.17

15

# TIGIT

15

*p* = 0.39

# VSIR

15

*p* = 0.22

# BTLA

15

*p* = 0.35

# IDO1

15

10 10 10 10 10

Expression

Expression

Expression

Expression

Expression

5 5 5 5 5

0 0 0 0 0

# CD276

*p* = 0.52

15

# VTCN1

15

*p* = 0.5

*GEM*

15

*p* = 0.39

*p* = 0.05

*p* = 0.35

10 10 10

Expression

Expression

Expression

5 5 5

0 0 0

# CD274

*p* = 0.026

15

# PDCD1LG2

15

*p* = 0.05

# PDCD1

15

*p* = 0.05

# CTLA4

15

*p* = 0.16

# HAVCR2

15

10 10 10 10 10

Expression

Expression

Expression

Expression

Expression

5 5 5 5 5

0 0 0 0 0

# LAG3

*p* = 0.026

15

# TIGIT

15

*p* = 0.071

# VSIR

15

*p* = 0.042

# BTLA

15

*p* = 0.24

# IDO1

15

10 10 10 10 10

Expression

Expression

Expression

Expression

Expression

5 5 5 5 5

0 0 0 0 0

# CD276

*p* = 0.12

15

# VTCN1

15

*p* = 0.64

*GEM*

15

*p* = 0.15

*p* = 0.042

*p* = 0.026

10 10 10

Expression

Expression

Expression

5 5 5

0 0 0

# CD274

*p* = 0.19

15

# PDCD1LG2

15

*p* = 0.77

# PDCD1

15

*p* = 0.1

# CTLA4

15

*p* = 0.032

# HAVCR2

15

10 10 10 10 10

Expression

Expression

Expression

Expression

Expression

5 5 5 5 5

0 0 0 0 0

# LAG3

*p* = 0.044

15

# TIGIT

15

*p* = 0.032

# VSIR

15

*p* = 0.032

# BTLA

15

*p* = 0.28

# IDO1

15

10 10 10 10 10

Expression

Expression

Expression

Expression

Expression

5 5 5 5 5

0 0 0 0 0

# CD276

*p* = 0.38

15

# VTCN1

15

*p* = 0.96

*GEM*

15

*p* = 0.85

*p* = 0.022

*p* = 0.044

10 10 10

Expression

Expression

Expression

5 5 5

0 0 0

# CD274

*p* = 0.0038

15

# PDCD1LG2

15

*p* = 0.052

# PDCD1

15

*p* = 0.18

# CTLA4

15

*p* = 0.097

# HAVCR2

15

10 10 10 10 10

Expression

Expression

Expression

Expression

Expression

5 5 5 5 5

0 0 0 0 0

# LAG3

*p* = 0.097

15

# TIGIT

15

*p* = 0.073

# VSIR

15

*p* = 0.073

# BTLA

15

*p* = 0.097

# IDO1

15

10 10 10 10 10

Expression

Expression

Expression

Expression

Expression

5 5 5 5 5

0 0 0 0 0

# CD276

*p* = 0.42

15

# VTCN1

15

*p* = 0.42

*GEM*

15

*p* = 0.12

*p* = 0.064

*p* = 0.32

10 10 10

Expression

Expression

Expression

5 5 5

0 0 0

# CD274

*p* = 0.94

15

# PDCD1LG2

15

*p* = 0.54

# PDCD1

15

*p* = 0.2

# CTLA4

15

*p* = 0.2

# HAVCR2

15

10 10 10 10 10

Expression

Expression

Expression

Expression

Expression

5 5 5 5 5

0 0 0 0 0

# LAG3

*p* = 0.37

15

# TIGIT

15

*p* = 0.2

# VSIR

15

*p* = 0.2

# BTLA

15

*p* = 0.7

# IDO1

15

10 10 10 10 10

Expression

Expression

Expression

Expression

Expression

5 5 5 5 5

0 0 0 0 0

# CD276

*p* = 0.94

15

# VTCN1

15

*p* = 0.94

*GEM*

15

*p* = 0.2

*p* = 0.2

*p* = 0.7

10 10 10

Expression

Expression

Expression

5 5 5

0 0 0

# CD274

*p* = 0.041

15

# PDCD1LG2

15

*p* = 0.054

# CTLA4

15

*p* = 0.065

10 10 10

Expression

Expression

Expression

5 5 5

0 0 0

# CD276

*p* = 0.38

15

# VTCN1

15

*p* = 0.47

*GEM*

15

*p* = 0.38

10 10 10

Expression

Expression

Expression

5 5 5

0 0 0

# PDCD1LG2

*p* = 0.093

15

# PDCD1

15

*p* = 0.37

# CTLA4

15

*p* = 0.22

10 10 10

Expression

Expression

Expression

5 5 5

0 0 0

# LAG3

*p* = 0.22

15

# IDO1

15

10 10

Expression

Expression

5 5

0 0

# VTCN1

*p* = 0.6

15

*GEM*

15

*p* = 0.22

*p* = 0.22

10 10

Expression

Expression

5 5

0 0

# PDCD1LG2

*p* = 0.83

15

# PDCD1

15

*p* = 0.16

# CTLA4

15

*p* = 0.52

10 10 10

Expression

Expression

Expression

5 5 5

0 0 0

# LAG3

*p* = 0.2

15

# IDO1

15

10 10

Expression

Expression

5 5

0 0

# VTCN1

*p* = 0.72

15

*GEM*

15

*p* = 0.16

*p* = 0.52

10 10

Expression

Expression

5 5

0 0

# PDCD1LG2

*p* = 0.23

15

# PDCD1

15

*p* = 0.089

# CTLA4

15

*p* = 0.05

10 10 10

Expression

Expression

Expression

5 5 5

0 0 0

# LAG3

*p* = 0.24

15

# IDO1

15

10 10

Expression

Expression

5 5

0 0

# VTCN1

*p* = 0.16

15

*GEM*

15

*p* = 0.16

*p* = 0.24

10 10

Expression

Expression

5 5

0 0

# CD274

*p* = 0.71

15

# PDCD1LG2

15

*p* = 0.37

# PDCD1

15

*p* = 0.23

# CTLA4

15

*p* = 0.38

# HAVCR2

15

10 10 10 10 10

Expression

Expression

Expression

Expression

Expression

5 5 5 5 5

0 0 0 0 0

# LAG3

*p* = 0.4

15

# TIGIT

15

*p* = 0.23

# VSIR

15

*p* = 0.23

# BTLA

15

*p* = 0.23

# IDO1

15

10 10 10 10 10

Expression

Expression

Expression

Expression

Expression

5 5 5 5 5

0 0 0 0 0

# CD276

*p* = 0.93

15

# VTCN1

15

*p* = 0.37

*GEM*

15

*p* = 0.25

*p* = 0.25

*p* = 0.72

10 10 10

Expression

Expression

Expression

5 5 5

0 0 0

# PDCD1LG2

*p* = 0.07

15

# PDCD1

15

*p* = 0.15

# CTLA4

15

*p* = 0.66

10 10 10

Expression

Expression

Expression

5 5 5

0 0 0

# LAG3

*p* = 0.15

15

# IDO1

15

10 10

Expression

Expression

5 5

0 0

# VTCN1

*p* = 0.89

15

*GEM*

15

*p* = 0.88

*p* = 0.07

10 10

Expression

Expression

5 5

0 0

# CD274

*p* = 0.68

15

# PDCD1LG2

15

*p* = 0.68

# CTLA4

15

*p* = 0.68

# HAVCR2

15

10 10 10 10

Expression

Expression

Expression

Expression

5 5 5 5

0 0 0 0

# LAG3

*p* = 0.68

15

# TIGIT

15

*p* = 0.79

# VSIR

15

*p* = 0.86

# BTLA

15

*p* = 0.68

# IDO1

15

10 10 10 10 10

Expression

Expression

Expression

Expression

Expression

5 5 5 5 5

0 0 0 0 0

# CD276

*p* = 0.68

15

# VTCN1

15

*p* = 0.68

*GEM*

15

*p* = 0.68

*p* = 0.68

*p* = 0.79

10 10 10

Expression

Expression

Expression

5 5 5

0 0 0

# CD274

*p* = 0.28

15

# PDCD1LG2

15

*p* = 0.42

# CTLA4

15

*p* = 0.4

# HAVCR2

15

10 10 10 10

Expression

Expression

Expression

Expression

5 5 5 5

0 0 0 0

# LAG3

*p* = 0.56

15

# TIGIT

15

*p* = 0.56

# VSIR

15

*p* = 0.56

# BTLA

15

*p* = 0.4

# IDO1

15

10 10 10 10 10

Expression

Expression

Expression

Expression

Expression

5 5 5 5 5

0 0 0 0 0

# CD276

*p* = 0.56

15

# VTCN1

15

*p* = 0.56

*GEM*

15

*p* = 0.46

*p* = 0.42

*p* = 0.4

10 10 10

Expression

Expression

Expression

5 5 5

0 0 0

# CD274

*p* = 0.89

15

# PDCD1LG2

15

*p* = 0.41

# PDCD1

15

*p* = 0.41

# CTLA4

15

*p* = 0.41

# HAVCR2

15

10 10 10 10 10

Expression

Expression

Expression

Expression

Expression

5 5 5 5 5

0 0 0 0 0

# LAG3

*p* = 0.41

15

# TIGIT

15

*p* = 0.94

# VSIR

15

*p* = 0.41

# BTLA

15

*p* = 0.65

# IDO1

15

10 10 10 10 10

Expression

Expression

Expression

Expression

Expression

5 5 5 5 5

0 0 0 0 0

# CD276

*p* = 0.94

15

# VTCN1

15

*p* = 0.94

*GEM*

15

*p* = 0.74

*p* = 0.41

*p* = 0.77

10 10 10

Expression

Expression

Expression

5 5 5

0 0 0

# PDCD1

*p* = 0.21

15

10

Expression

5

0

# LAG3

*p* = 0.024

15

# IDO1

15

*p* = 0.024

10 10

Expression

Expression

5 5

0 0

# GEM

*p* = 0.35

15

10

Expression

5

0

# CD274

*p* = 0.18

15

# PDCD1LG2

15

*p* = 0.15

# PDCD1

15

*p* = 0.15

# CTLA4

15

*p* = 0.42

# HAVCR2

15

10 10 10 10 10

Expression

Expression

Expression

Expression

Expression

5 5 5 5 5

0 0 0 0 0

# LAG3

*p* = 0.24

15

# TIGIT

15

*p* = 0.15

# VSIR

15

*p* = 0.18

# BTLA

15

*p* = 0.52

# IDO1

15

10 10 10 10 10

Expression

Expression

Expression

Expression

Expression

5 5 5 5 5

0 0 0 0 0

# CD276

*p* = 0.18

15

# VTCN1

15

*p* = 0.24

*GEM*

15

*p* = 0.24

*p* = 0.15

*p* = 0.15

10 10 10

Expression

Expression

Expression

5 5 5

0 0 0

# CD274

*p* = 0.35

15

# PDCD1LG2

15

*p* = 0.22

# PDCD1

15

*p* = 0.56

# CTLA4

15

*p* = 0.22

# HAVCR2

15

10 10 10 10 10

Expression

Expression

Expression

Expression

Expression

5 5 5 5 5

0 0 0 0 0

# LAG3

*p* = 0.22

15

# TIGIT

15

*p* = 0.22

# VSIR

15

*p* = 0.22

# BTLA

15

*p* = 0.22

# IDO1

15

10 10 10 10 10

Expression

Expression

Expression

Expression

Expression

5 5 5 5 5

0 0 0 0 0

# CD276

*p* = 0.22

15

# VTCN1

15

*p* = 0.22

*GEM*

15

*p* = 0.22

*p* = 0.22

*p* = 0.23

10 10 10

Expression

Expression

Expression

5 5 5

0 0 0

# CD274

*p* = 0.77

15

# CTLA4

15

*p* = 0.77

10 10

Expression

Expression

5 5

0 0

# VSIR

*p* = 0.65

15

# BTLA

15

*p* = 0.65

# IDO1

15

10 10 10

Expression

Expression

Expression

5 5 5

0 0 0

# CD276

*p* = 0.77

15

# VTCN1

15

*p* = 0.73

*GEM*

15

*p* = 0.65

*p* = 0.65

10 10 10

Expression

Expression

Expression

5 5 5

0 0 0

# PDCD1LG2

*p* = 0.29

15

# PDCD1

15

*p* = 0.33

# CTLA4

15

*p* = 0.35

10 10 10

Expression

Expression

Expression

5 5 5

0 0 0

# LAG3

*p* = 0.14

15

# IDO1

15

*p* = 0.33

10 10

Expression

Expression

5 5

0 0

# GEM

*p* = 0.33

15

10

Expression

5

0

# CD274

*p* = 0.54

15

# PDCD1LG2

15

*p* = 0.54

# PDCD1

15

*p* = 0.65

# CTLA4

15

*p* = 0.9

# HAVCR2

15

10 10 10 10 10

Expression

Expression

Expression

Expression

Expression

5 5 5 5 5

0 0 0 0 0

# LAG3

*p* = 0.76

15

# TIGIT

15

*p* = 0.82

# VSIR

15

*p* = 0.54

# BTLA

15

*p* = 0.91

# IDO1

15

10 10 10 10 10

Expression

Expression

Expression

Expression

Expression

5 5 5 5 5

0 0 0 0 0

# CD276

*p* = 0.54

15

# VTCN1

15

*p* = 0.54

*GEM*

15

*p* = 0.72

*p* = 0.54

*p* = 0.54

10 10 10

Expression

Expression

Expression

5 5 5

0 0 0

# CD274

*p* = 0.28

15

# PDCD1LG2

15

*p* = 0.2

# PDCD1

15

*p* = 0.2

# CTLA4

15

*p* = 0.2

# HAVCR2

15

10 10 10 10 10

Expression

Expression

Expression

Expression

Expression

5 5 5 5 5

0 0 0 0 0

# LAG3

*p* = 0.3

15

# TIGIT

15

*p* = 0.2

# VSIR

15

*p* = 0.28

# BTLA

15

*p* = 0.2

# IDO1

15

10 10 10 10 10

Expression

Expression

Expression

Expression

Expression

5 5 5 5 5

0 0 0 0 0

# CD276

*p* = 0.2

15

# VTCN1

15

*p* = 0.43

*GEM*

15

*p* = 0.91

*p* = 0.28

*p* = 0.43

10 10 10

Expression

Expression

Expression

5 5 5

0 0 0

# PDCD1

*p* = 0.46

15

10

Expression

5

0

# LAG3

*p* = 0.46

15

# IDO1

15

*p* = 0.81

10 10

Expression

Expression

5 5

0 0

# GEM

*p* = 0.46

15

10

Expression

5

0

# CD274

*p* = 0.37

15

# PDCD1LG2

15

*p* = 0.37

# PDCD1

15

*p* = 0.37

# CTLA4

15

*p* = 0.52

# HAVCR2

15

10 10 10 10 10

Expression

Expression

Expression

Expression

Expression

5 5 5 5 5

0 0 0 0 0

# LAG3

*p* = 0.79

15

# TIGIT

15

*p* = 0.79

# VSIR

15

*p* = 0.37

# BTLA

15

*p* = 0.69

# IDO1

15

10 10 10 10 10

Expression

Expression

Expression

Expression

Expression

5 5 5 5 5

0 0 0 0 0

# CD276

*p* = 0.37

15

# VTCN1

15

*p* = 0.37

*GEM*

15

*p* = 0.37

*p* = 0.37

*p* = 0.78

10 10 10

Expression

Expression

Expression

5 5 5

0 0 0

# CD274

*p* = 0.37

15

# PDCD1LG2

15

*p* = 0.49

# PDCD1

15

*p* = 0.37

# CTLA4

15

*p* = 0.37

# HAVCR2

15

10 10 10 10 10

Expression

Expression

Expression

Expression

Expression

5 5 5 5 5

0 0 0 0 0

# LAG3

*p* = 0.76

15

# TIGIT

15

*p* = 0.49

# VSIR

15

*p* = 0.37

# BTLA

15

*p* = 0.43

# IDO1

15

10 10 10 10 10

Expression

Expression

Expression

Expression

Expression

5 5 5 5 5

0 0 0 0 0

# CD276

*p* = 0.91

15

# VTCN1

15

*p* = 0.91

*GEM*

15

*p* = 0.37

*p* = 0.37

*p* = 0.37

10 10 10

Expression

Expression

Expression

5 5 5

0 0 0

**Supplementary Fig. 8. Relative activity levels of the fifty studied pathways in each of the 111 CPTAC-3 LUAD samples that were assigned to a consensus subtype.** Red colors indicate higher relative activity of a pathway in a certain sample, whereas blue colors indicate lower relative activity of a pathway in a certain sample.

## Supplementary Fig. 9. LUAD cancer cell lines (LUAD-CCL) used for the potential treatment strategies discovery analysis. (Next page)

### Summary of mutated or altered genes for CCLE project cell lines (CTRPv2 and PRISM drug sensitivity datasets).

1. Summary of mutated or altered genes for GDSC project cell lines (GDSC drug sensitivity datasets).

### Cell lines in each dataset were classified into the different LUAD subtypes using the predict function of the UMAP R package based on the global behavior of the same 50 pathways used for the primary tumors’ classification. Genomic characteristics regarding important driver genes for NSCLC clinical management are displayed. Green color represents the presence of a mutation in a specific gene. Cell line names in each subtype are named after their Cellosaurus ID.
