# Supplementary material for: Transcriptional analysis of landmark molecular pathways in lung adenocarcinoma results in a clinically relevant classification with potential therapeutic implications
Source: Mol Oncol. 2023 Dec 21;18(2):453–70. doi: 10.1002/1878-0261.13550 (PMC10850798; doi:10.1002/1878-0261.13550)
Supplement: Supplementary file 2 — Table S1. List of gene expression datasets included in this study. Table S2. Correlation of dataset ids with LUAD subtypes. Table S3. FDR‐adjusted p values for pairwise comparisons of TMB values between LUAD subtypes. Table S4. Percentage of positive patients for each single nucleotide base substitution mutational signature across LUAD subtypes. Table S5. FDR‐adjusted p values for pairwise comparisons of copy number rate values between LUAD subtypes. Table S6. FDR‐adjusted p values for pairwise comparisons of copy number rate values between LUAD subtypes. [file MOL2-18-453-s002.docx]

**SUPPLEMENTARY TABLES**

**Supplementary Table 1.** List of gene expression datasets included in this study.

| **Dataset ID** | **Paper ID** | **Resource** | **Platform** | **Number LUAD samples** |
| --- | --- | --- | --- | --- |
| E-MEXP-231 | [15653641](https://www.ncbi.nlm.nih.gov/pubmed/15653641) | ArrayExpress (Yap) | Affymetrix HG-U133A | 48 |
| [E-MTAB-1132](https://www.ebi.ac.uk/arrayexpress/experiments/E-MTAB-1132) | [25944621](https://www.ncbi.nlm.nih.gov/pubmed/25944621) | ArrayExpress (Lazar) | Agilent_Human_244K_Exonv3 | 57 |
| [E-MTAB-1790](https://www.ebi.ac.uk/arrayexpress/experiments/E-MTAB-1790) | [25301404](https://www.ncbi.nlm.nih.gov/pubmed/25301404) | ArrayExpress (Sanz) | Agilent HG array 44k_014850 | 38 |
| [E-MTAB-5231](https://www.ebi.ac.uk/gxa/experiments/E-MTAB-5231?ref=aebrowse) | [28292941](https://www.ncbi.nlm.nih.gov/pubmed/28292941) | ArrayExpress (Joerg Mueller) | Affymetrix HG-U133_Plus_2 | 11 |
| E-MTAB-923 | [22914773](https://www.ncbi.nlm.nih.gov/pubmed/22914773) | ArrayExpress (Fouret) | Affymetrix HG-U133_Plus_2 | 103 |
| E-TABM-15 | - | ArrayExpress (Blum) | Affymetrix HG-U133A | 23 |
| E-TABM-185 | [20379172](https://www.ncbi.nlm.nih.gov/pubmed/20379172) | ArrayExpress | Affymetrix HG-U133A | 49 |
| GSE10072 | [18297132](https://www.ncbi.nlm.nih.gov/pubmed/18297132) | GEO (Landi) | Affymetrix HG-U133A | 58 |
| GSE10245 | [18486272](https://www.ncbi.nlm.nih.gov/pubmed/18486272) | GEO (Kuner) | Affymetrix HG-U133_Plus_2 | 34 |
| GSE1037 | [21737174](https://www.ncbi.nlm.nih.gov/pubmed/21737174) | GEO (Ishikawa) | CHUGAI 41K | 16 |
| GSE10445 | [19176396](https://www.ncbi.nlm.nih.gov/pubmed/19176396) | GEO (MERLION) | Affymetrix HG-U133_Plus_2 | 72 |
| GSE116959 | - | GEO (Rezzonico R) | Agilent-039494 SurePrint G3 Human GE v2 8x60K | 56 |
| GSE12236 | [18927117](https://www.ncbi.nlm.nih.gov/pubmed/18927117) | GEO (Xi) | Affymetrix HG 1.0 ST Exon Array | 20 |
| GSE12667 | [18948947](http://www.ncbi.nlm.nih.gov/pubmed/18948947) | GEO (Wilson) | Affymetrix HG-U133_Plus_2 | 68 |
| GSE13213 | [19414676](https://www.ncbi.nlm.nih.gov/pubmed/19414676) | GEO (Tomida) | Agilent_44k_014850 | 117 |
| GSE14814 | [20823422](https://www.ncbi.nlm.nih.gov/pubmed/20823422) | GEO (Zhu) | Affymetrix HG-U133A | 28 |
| [GSE16534](https://www.ncbi.nlm.nih.gov/geo/query/acc.cgi?acc=GSE16534) | [19737969](https://www.ncbi.nlm.nih.gov/pubmed/19737969) | GEO (Lin) | Affymetrix HG 1.0 ST Exon Array | 21 |
| GSE17475 | [20196851](https://www.ncbi.nlm.nih.gov/pubmed/20196851) | GEO (Neumann) | Affymetrix HG-U133A | 28 |
| [GSE18842](https://www.ncbi.nlm.nih.gov/geo/query/acc.cgi?acc=GSE18842) | [20878980](https://www.ncbi.nlm.nih.gov/pubmed/20878980) | GEO (Sánchez-Palencia) | Affymetrix HG-U133_Plus_2 | 14 |
| [GSE19188](https://www.ncbi.nlm.nih.gov/geo/query/acc.cgi?acc=GSE19188) | [20421987](https://www.ncbi.nlm.nih.gov/pubmed/20421987) | GEO (Hou) | Affymetrix HG-U133_Plus_2 | 45 |
| GSE20853 | [21737174](https://www.ncbi.nlm.nih.gov/pubmed/21737174) | GEO (Fujiwara) | CHUGAI 41K (spotted DNA/cDNA) | 154 |
| GSE2109 | - | GEO (expO) | Affymetrix HG-U133_Plus_2 | 54 |
| GSE2514 | [16314486](https://www.ncbi.nlm.nih.gov/pubmed/16314486) | GEO (Stearman) | Affymetrix HG_U95Av2 | 20 |
| GSE27262 | [22726390](https://www.ncbi.nlm.nih.gov/pubmed/22726390) | GEO (Wei) | Affymetrix HG-U133_Plus_2 | 25 |
| GSE27716 | [21911454](https://www.ncbi.nlm.nih.gov/pubmed/21911454) | GEO (Powell) | Affymetrix HG-U133_Plus_2 | 40 |
| GSE28571 | [22011649](https://www.ncbi.nlm.nih.gov/pubmed/22011649) | GEO (Botling) | Affymetrix HG-U133_Plus_2 | 50 |
| GSE28827 | [22710433](https://www.ncbi.nlm.nih.gov/pubmed/22710433) | GEO (Plevritis) | Illumina HumanHT-12 v3.0 | 20 |
| [GSE29016](https://www.ncbi.nlm.nih.gov/geo/query/acc.cgi?acc=GSE29016) | [22676229](https://www.ncbi.nlm.nih.gov/pubmed/22676229) | GEO (Staaf) | Illumina HumanHT-12 v3.0 | 38 |
| GSE30219 | [23698379](https://www.ncbi.nlm.nih.gov/pubmed/23698379) | GEO (Rousseaux) | Affymetrix HG-U133_Plus_2 | 84 |
| GSE31210 | [22080568](https://www.ncbi.nlm.nih.gov/pubmed/?term=22080568) | GEO (Yokota) | Affymetrix HG-U133_Plus_2 | 204 |
| GSE3141 | [16273092](https://www.ncbi.nlm.nih.gov/pubmed/16273092) | GEO (Bild) | Affymetrix HG-U133_Plus_2 | 58 |
| GSE31552 | [25128906](https://www.ncbi.nlm.nih.gov/pubmed/25128906) | GEO (Spivack) | Affymetrix HG 1.0 ST Gene Array | 19 |
| GSE31799 | [21911935](https://www.ncbi.nlm.nih.gov/pubmed/21911935) | GEO (Starczynowski) | Affymetrix Custom Rosetta Human platform | 29 |
| GSE31908 | [25335090](https://www.ncbi.nlm.nih.gov/pubmed/25335090) | GEO (Zhang) | Affymetrix HG-U133A + HG-U133B | 47 |
| GSE32665 | [23591868](https://www.ncbi.nlm.nih.gov/pubmed/23591868) | GEO (Kim I) | Illumina human-6 v2.0 expression beadchip | 87 |
| GSE32863 | [22613842](https://www.ncbi.nlm.nih.gov/pubmed/22613842) | GEO (Laird-Offringa) | Illumina HumanWG-6 v3.0 | 58 |
| GSE33072 | [23091115](https://www.ncbi.nlm.nih.gov/pubmed/23091115) | GEO (BATTLE_trial) | Affymetrix HG 1.0 ST Gene Array | 14 |
| GSE33532 | - | GEO (Meister) | Affymetrix HG-U133_Plus_2 | 64 |
| GSE37745 | [23032747](https://www.ncbi.nlm.nih.gov/pubmed/23032747) | GEO (Botling) | Affymetrix HG-U133_Plus_2 | 106 |
| GSE41271 | [23449933](https://www.ncbi.nlm.nih.gov/pubmed/23449933) | GEO (Sato) | Illumina HumanWG-6 v3.0 | 183 |
| GSE42127 | [23357979](https://www.ncbi.nlm.nih.gov/pubmed/23357979) | GEO (Tang) | Illumina HumanWG-6 v3.0 | 133 |
| GSE43458 | [23659968](https://www.ncbi.nlm.nih.gov/pubmed/23659968) | GEO (Kabbout) | Affymetrix HG 1.0 ST Gene Array | 80 |
| GSE43580 | [23966112](https://www.ncbi.nlm.nih.gov/pubmed/23966112) | GEO (Talikka M) | Affymetrix Human Genome U133 Plus 2.0 Array | 75 |
| GSE50081 | [24305008](https://www.ncbi.nlm.nih.gov/pubmed/24305008) | GEO (Der) | Affymetrix HG-U133_Plus_2 | 128 |
| GSE6044 | [18992152](https://www.ncbi.nlm.nih.gov/pubmed/18992152) | GEO (Rohr) | Affymetrix Human HG-Focus Target Array | 16 |
| GSE60644 | [25278450](https://www.ncbi.nlm.nih.gov/pubmed/25278450) | GEO (Karlsson A) | Illumina HumanHT-12 V4.0 expression beadchip | 77 |
| GSE6253 | [17194181](https://www.ncbi.nlm.nih.gov/pubmed/17194181) | GEO (Lu) | Affymetrix HG U95A Array | 14 |
| GSE66759 | [26483346](https://www.ncbi.nlm.nih.gov/pubmed/26483346) | GEO (Takahashi) | Agilent Human GE 8x60k_028004 | 76 |
| GSE66863 | [26601720](https://www.ncbi.nlm.nih.gov/pubmed/26601720) | GEO (Bjaanaes) | Agilent Human GE 8x60k_028004 | 120 |
| GSE68465 | [18641660](https://www.ncbi.nlm.nih.gov/pubmed/18641660) | GEO (Shedden) | Affymetrix HG-U133A | 443 |
| GSE72094 | [26477306](https://www.ncbi.nlm.nih.gov/pubmed/26477306) | GEO ( Schabath M) | Rosetta/Merck Human RSTA Custom Affymetrix 2.0 microarray | 442 |
| GSE74706 | [27197161](https://www.ncbi.nlm.nih.gov/pubmed/27197161) | GEO (Marwitz S) | Agilent-026652 Whole Human Genome Microarray 4x44K v2 | 10 |
| GSE7670 | [17540040](https://www.ncbi.nlm.nih.gov/pubmed/17540040) | GEO (Su) | Affymetrix HG-U133A | 26 |
| GSE8894 | [19010856](https://www.ncbi.nlm.nih.gov/pubmed/19010856) | GEO (Lee) | Affymetrix HG-U133_Plus_2 | 63 |
| [cBio@MSKCC](http://cbio.mskcc.org/public/lung_array_data/) | [19525976](https://www.ncbi.nlm.nih.gov/pubmed/19525976) | MSKCC (Chitale) | Affymetrix HG-U133A_2 | 102 |
| TCGA-LUAD | [25079552](https://www.ncbi.nlm.nih.gov/pubmed/25079552) | cBioPortal | RNA-Seq (Illumina HiSeq 2000) | 515 |
| **Total** |  |  |  | **4580** |

**Supplementary Table 2. Correlation of dataset ids with LUAD subtypes.**

|  | **AD1** | **AD2** | **AD3** | **AD4** | **AD5** | **AD6** | **AD7** |  |
| --- | --- | --- | --- | --- | --- | --- | --- | --- |
|  | N=770 | N=855 | N=477 | N=603 | N=958 | N=779 | N=131 | p |
| **Dataset, N (%):** |  |  |  |  |  |  |  | <0.001 |
| Bild | 3 (0.39%) | 12 (1.40%) | 7 (1.47%) | 13 (2.16%) | 11 (1.15%) | 10 (1.28%) | 2 (1.53%) |  |
| Botling | 16 (2.08%) | 26 (3.04%) | 14 (2.94%) | 10 (1.66%) | 21 (2.19%) | 16 (2.05%) | 3 (2.29%) |  |
| Der | 19 (2.47%) | 18 (2.11%) | 21 (4.40%) | 20 (3.32%) | 30 (3.13%) | 15 (1.93%) | 5 (3.82%) |  |
| Ding | 14 (1.82%) | 14 (1.64%) | 9 (1.89%) | 4 (0.66%) | 13 (1.36%) | 12 (1.54%) | 2 (1.53%) |  |
| E-MEXP-231 | 5 (0.65%) | 9 (1.05%) | 7 (1.47%) | 10 (1.66%) | 10 (1.04%) | 6 (0.77%) | 0 (0.00%) |  |
| E-MTAB-1132 | 9 (1.17%) | 11 (1.29%) | 4 (0.84%) | 11 (1.82%) | 10 (1.04%) | 9 (1.16%) | 3 (2.29%) |  |
| E-MTAB-1790 | 9 (1.17%) | 11 (1.29%) | 1 (0.21%) | 2 (0.33%) | 10 (1.04%) | 5 (0.64%) | 0 (0.00%) |  |
| E-MTAB-5231 | 3 (0.39%) | 2 (0.23%) | 0 (0.00%) | 1 (0.17%) | 4 (0.42%) | 1 (0.13%) | 0 (0.00%) |  |
| E-MTAB-923 | 20 (2.60%) | 20 (2.34%) | 8 (1.68%) | 8 (1.33%) | 25 (2.61%) | 15 (1.93%) | 7 (5.34%) |  |
| E-TABM-15 | 3 (0.39%) | 3 (0.35%) | 2 (0.42%) | 7 (1.16%) | 4 (0.42%) | 4 (0.51%) | 0 (0.00%) |  |
| E-TABM-185 | 6 (0.78%) | 9 (1.05%) | 7 (1.47%) | 9 (1.49%) | 10 (1.04%) | 6 (0.77%) | 1 (0.76%) |  |
| expO | 7 (0.91%) | 8 (0.94%) | 11 (2.31%) | 9 (1.49%) | 11 (1.15%) | 8 (1.03%) | 0 (0.00%) |  |
| Fujiwara | 36 (4.68%) | 15 (1.75%) | 7 (1.47%) | 19 (3.15%) | 23 (2.40%) | 32 (4.11%) | 22 (16.79%) |  |
| GSE10445 | 4 (0.52%) | 18 (2.11%) | 14 (2.94%) | 12 (1.99%) | 14 (1.46%) | 10 (1.28%) | 0 (0.00%) |  |
| GSE116959 | 11 (1.43%) | 13 (1.52%) | 11 (2.31%) | 4 (0.66%) | 9 (0.94%) | 6 (0.77%) | 2 (1.53%) |  |
| GSE27262 | 5 (0.65%) | 5 (0.58%) | 4 (0.84%) | 2 (0.33%) | 5 (0.52%) | 3 (0.39%) | 1 (0.76%) |  |
| GSE27716 | 3 (0.39%) | 4 (0.47%) | 7 (1.47%) | 11 (1.82%) | 1 (0.10%) | 11 (1.41%) | 3 (2.29%) |  |
| GSE28827 | 5 (0.65%) | 2 (0.23%) | 1 (0.21%) | 3 (0.50%) | 5 (0.52%) | 3 (0.39%) | 1 (0.76%) |  |
| GSE31552 | 2 (0.26%) | 3 (0.35%) | 2 (0.42%) | 5 (0.83%) | 2 (0.21%) | 5 (0.64%) | 0 (0.00%) |  |
| GSE31799 | 6 (0.78%) | 4 (0.47%) | 3 (0.63%) | 2 (0.33%) | 8 (0.84%) | 6 (0.77%) | 0 (0.00%) |  |
| GSE31908 | 9 (1.17%) | 6 (0.70%) | 6 (1.26%) | 6 (1.00%) | 10 (1.04%) | 8 (1.03%) | 2 (1.53%) |  |
| GSE32665 | 25 (3.25%) | 11 (1.29%) | 2 (0.42%) | 5 (0.83%) | 18 (1.88%) | 22 (2.82%) | 4 (3.05%) |  |
| GSE33072 | 2 (0.26%) | 0 (0.00%) | 4 (0.84%) | 3 (0.50%) | 1 (0.10%) | 4 (0.51%) | 0 (0.00%) |  |
| GSE33532 | 7 (0.91%) | 12 (1.40%) | 7 (1.47%) | 7 (1.16%) | 16 (1.67%) | 14 (1.80%) | 1 (0.76%) |  |
| GSE43580 | 9 (1.17%) | 20 (2.34%) | 2 (0.42%) | 3 (0.50%) | 22 (2.30%) | 17 (2.18%) | 2 (1.53%) |  |
| GSE6044 | 2 (0.26%) | 2 (0.23%) | 0 (0.00%) | 3 (0.50%) | 4 (0.42%) | 4 (0.51%) | 1 (0.76%) |  |
| GSE60644 | 17 (2.21%) | 13 (1.52%) | 3 (0.63%) | 3 (0.50%) | 23 (2.40%) | 14 (1.80%) | 3 (2.29%) |  |
| GSE6253_GPL96 | 1 (0.13%) | 3 (0.35%) | 2 (0.42%) | 3 (0.50%) | 2 (0.21%) | 3 (0.39%) | 0 (0.00%) |  |
| GSE66759 | 15 (1.95%) | 15 (1.75%) | 3 (0.63%) | 6 (1.00%) | 18 (1.88%) | 16 (2.05%) | 3 (2.29%) |  |
| GSE66863 | 15 (1.95%) | 25 (2.92%) | 16 (3.35%) | 11 (1.82%) | 29 (3.03%) | 20 (2.57%) | 4 (3.05%) |  |
| GSE68465 | 62 (8.05%) | 72 (8.42%) | 64 (13.42%) | 87 (14.43%) | 70 (7.31%) | 83 (10.65%) | 5 (3.82%) |  |
| GSE74706 | 0 (0.00%) | 3 (0.35%) | 0 (0.00%) | 1 (0.17%) | 4 (0.42%) | 2 (0.26%) | 0 (0.00%) |  |
| Hou | 3 (0.39%) | 13 (1.52%) | 5 (1.05%) | 6 (1.00%) | 11 (1.15%) | 7 (0.90%) | 0 (0.00%) |  |
| Jones | 3 (0.39%) | 4 (0.47%) | 1 (0.21%) | 1 (0.17%) | 5 (0.52%) | 2 (0.26%) | 0 (0.00%) |  |
| Kabbout | 10 (1.30%) | 13 (1.52%) | 14 (2.94%) | 15 (2.49%) | 12 (1.25%) | 16 (2.05%) | 0 (0.00%) |  |
| Kuner | 4 (0.52%) | 9 (1.05%) | 4 (0.84%) | 4 (0.66%) | 8 (0.84%) | 5 (0.64%) | 0 (0.00%) |  |
| Landi | 8 (1.04%) | 9 (1.05%) | 8 (1.68%) | 13 (2.16%) | 11 (1.15%) | 8 (1.03%) | 1 (0.76%) |  |
| Lee | 5 (0.65%) | 12 (1.40%) | 9 (1.89%) | 11 (1.82%) | 13 (1.36%) | 11 (1.41%) | 2 (1.53%) |  |
| Lin | 3 (0.39%) | 1 (0.12%) | 1 (0.21%) | 4 (0.66%) | 4 (0.42%) | 8 (1.03%) | 0 (0.00%) |  |
| Micke | 7 (0.91%) | 13 (1.52%) | 4 (0.84%) | 8 (1.33%) | 12 (1.25%) | 5 (0.64%) | 1 (0.76%) |  |
| MSKCC | 20 (2.60%) | 18 (2.11%) | 11 (2.31%) | 13 (2.16%) | 20 (2.09%) | 19 (2.44%) | 1 (0.76%) |  |
| Neumann | 3 (0.39%) | 3 (0.35%) | 5 (1.05%) | 5 (0.83%) | 4 (0.42%) | 7 (0.90%) | 1 (0.76%) |  |
| Okayama | 52 (6.75%) | 24 (2.81%) | 15 (3.14%) | 22 (3.65%) | 43 (4.49%) | 43 (5.52%) | 5 (3.82%) |  |
| Rousseaux | 18 (2.34%) | 17 (1.99%) | 9 (1.89%) | 9 (1.49%) | 17 (1.77%) | 11 (1.41%) | 3 (2.29%) |  |
| Sanchez-Palencia | 4 (0.52%) | 2 (0.23%) | 2 (0.42%) | 0 (0.00%) | 4 (0.42%) | 2 (0.26%) | 0 (0.00%) |  |
| Sato | 37 (4.81%) | 39 (4.56%) | 16 (3.35%) | 21 (3.48%) | 36 (3.76%) | 27 (3.47%) | 7 (5.34%) |  |
| Schabath | 74 (9.61%) | 89 (10.41%) | 44 (9.22%) | 52 (8.62%) | 110 (11.48%) | 61 (7.83%) | 10 (7.63%) |  |
| Selamat | 13 (1.69%) | 12 (1.40%) | 2 (0.42%) | 3 (0.50%) | 11 (1.15%) | 15 (1.93%) | 2 (1.53%) |  |
| Staaf | 9 (1.17%) | 8 (0.94%) | 3 (0.63%) | 2 (0.33%) | 11 (1.15%) | 4 (0.51%) | 1 (0.76%) |  |
| Stearman | 4 (0.52%) | 4 (0.47%) | 4 (0.84%) | 4 (0.66%) | 3 (0.31%) | 1 (0.13%) | 0 (0.00%) |  |
| Su | 2 (0.26%) | 2 (0.23%) | 6 (1.26%) | 8 (1.33%) | 1 (0.10%) | 6 (0.77%) | 1 (0.76%) |  |
| Tang | 27 (3.51%) | 28 (3.27%) | 12 (2.52%) | 17 (2.82%) | 26 (2.71%) | 16 (2.05%) | 7 (5.34%) |  |
| TCGA-LUAD | 90 (11.69%) | 114 (13.33%) | 34 (7.13%) | 66 (10.95%) | 113 (11.80%) | 85 (10.91%) | 12 (9.16%) |  |
| Tomida | 19 (2.47%) | 22 (2.57%) | 10 (2.10%) | 12 (1.99%) | 30 (3.13%) | 23 (2.95%) | 0 (0.00%) |  |
| Xi | 4 (0.52%) | 4 (0.47%) | 3 (0.63%) | 1 (0.17%) | 4 (0.42%) | 4 (0.51%) | 0 (0.00%) |  |
| Zhu | 1 (0.13%) | 6 (0.70%) | 6 (1.26%) | 6 (1.00%) | 6 (0.63%) | 3 (0.39%) | 0 (0.00%) |  |

**Supplementary Table 3.** FDR-adjusted p values for pairwise comparisons of TMB values between LUAD subtypes. Significant p values are marked in bold.

|  | **AD1** | **AD2** | **AD3** | **AD4** | **AD5** | **AD6** |
| --- | --- | --- | --- | --- | --- | --- |
| **AD2** | **6.8e-10** | - | - | - |  |  |
| **AD3** | 0.31727 | **0.00089** | - | - |  |  |
| **AD4** | 0.13857 | **7.7e-09** | 0.14129 | - |  |  |
| **AD5** | 0.28123 | **1.9e-10** | 0.15161 | 0.81430 |  |  |
| **AD6** | **4.1e-08** | 0.38188 | **0.01065** | **1.4e-07** | **2.0e-08** |  |
| **AD7** | 0.16628 | 0.66330 | 0.32809 | 0.11673 | 0.12514 | 0.82654 |

**Supplementary Table 4**. Percentage of positive patients for each single nucleotide base substitution mutational signature across LUAD subtypes. p values come from a proportions test adjusted by FDR. Significant p values are marked in bold.

|  | **AD1** | **AD2** | **AD3** | **AD4** | **AD5** | **AD6** | **AD7** | ***p*** |
| --- | --- | --- | --- | --- | --- | --- | --- | --- |
| **Total *N*** | 90 | 111 | 34 | 66 | 111 | 84 | 11 |  |
| SBS1 (clock−like) | 95.96 | 99.10 | 100 | 86.36 | 92.79 | 97.62 | 100 | **6.76e-03** |
| SBS2 (APOBEC activity) | 34.44 | 37.96 | 51.52 | 31.25 | 28.83 | 49.40 | 45.45 | 6.26e-02 |
| SBS4 (Tobacco smoking) | 74.16 | 83.02 | 58.82 | 57.81 | 66.06 | 85.54 | 72.73 | **1.52e-03** |
| SBS5 (clock−like) | 98.89 | 99.07 | 100 | 96.92 | 96.40 | 98.75 | 100 | 6.32e-01 |
| SBS13 (APOBEC activity) | 26.67 | 37.04 | 48.48 | 27.69 | 25.23 | 49.40 | 40 | **6.76e-03** |
| SBS15 (MMR deficiency) | 15.56 | 5.41 | 2.94 | 18.18 | 9.91 | 8.43 | 9.09 | 6.50e-02 |

**Supplementary Table 5.** FDR-adjusted p values for pairwise comparisons of copy number rate values between LUAD subtypes. Significant p values are marked in bold.

|  | **AD1** | **AD2** | **AD3** | **AD4** | **AD5** | **AD6** |
| --- | --- | --- | --- | --- | --- | --- |
| **AD2** | **1.8e-07** | - | - | - |  |  |
| **AD3** | 0.94281 | **5.8e-05** | - | - |  |  |
| **AD4** | 0.94281 | **1.4e-06** | 0.94281 | - |  |  |
| **AD5** | 0.05288 | **9.2e-16** | 0.12747 | 0.10143 |  |  |
| **AD6** | **0.00043** | 0.11284 | **0.00555** | **0.00097** | **2.3e-09** |  |
| **AD7** | 0.34950 | 0.23129 | 0.31212 | 0.34950 | 0.05283 | 0.65940 |

**Supplementary Table 6.** FDR-adjusted p values for pairwise comparisons of copy number rate values between LUAD subtypes. Significant p values are marked in bold.

|  | **AD1** | **AD2** | **AD3** | **AD4** | **AD5** | **AD6** |
| --- | --- | --- | --- | --- | --- | --- |
| **AD2** | **1.5e-12** | - | - | - |  |  |
| **AD3** | 0.3506 | **0.0482** | - | - |  |  |
| **AD4** | **0.0482** | **0.0022** | 0.7968 | - |  |  |
| **AD5** | 0.5093 | **2.9e-10** | 0.2377 | **0.0337** |  |  |
| **AD6** | **2.3e-08** | 0.6048 | 0.0912 | **0.0227** | **1.2e-07** |  |
| **AD7** | 0.0872 | 0.2377 | 0.7514 | 0.4595 | 0.0508 | 0.4595 |
